# A proteolytically activated antimicrobial toxin encoded on a mobile plasmid of Bacteroidales induces a protective response

**DOI:** 10.1038/s41467-022-31925-w

**Published:** 2022-07-23

**Authors:** Jordan C. Evans, Valentina Laclare McEneany, Michael J. Coyne, Elizabeth P. Caldwell, Madeline L. Sheahan, Salena S. Von, Emily M. Coyne, Rodney K. Tweten, Laurie E. Comstock

**Affiliations:** 1 Department of Microbiology and Immunology, University of Oklahoma Health Sciences Center, Oklahoma City, OK 73104 USA; 2 Division of Infectious Diseases, Brigham and Women’s Hospital/Harvard Medical School, 181 Longwood Avenue, Boston, MA 02115 USA; 3 Duchossois Family Institute and Department of Microbiology, University of Chicago, Chicago, IL USA

**Keywords:** Bacterial genetics, Microbial communities, Bacterial toxins

## Abstract

*Phocaeicola vulgatus* is one of the most abundant and ubiquitous bacterial species of the human gut microbiota, yet a comprehensive analysis of antibacterial toxin production by members of this species has not been reported. Here, we identify and characterize a previously undescribed antibacterial protein. This toxin, designated BcpT, is encoded on a small mobile plasmid that is largely confined to strains of the closely related species *Phocaeicola vulgatus* and *Phocaeicola dorei*. BcpT is unusual in that it requires cleavage at two distinct sites for activation, and we identify bacterial proteases that perform this activation. We further identify BcpT’s receptor as the Lipid A-core glycan, allowing BcpT to target species of other Bacteroidales families. Exposure of cells to BcpT induces a response involving an unusual sigma/anti-sigma factor pair that is likely triggered by cell envelope stress, resulting in the expression of genes that partially protect cells from multiple antimicrobial toxins.

## Abstract (web summary)

The bacterium *Phocaeicola vulgatus* is commonly found in the human gut. Here, the authors show that the microorganism produces an antibacterial toxin that targets the LPS core glycan of closely related species and induces a response that partially protects cells from multiple antimicrobial toxins.

## Introduction

Globally, species of the order Bacteroidales are the most abundant Gram-negative bacteria of the healthy gut microbiota. Competition for membership in this dense microbial community depends on many factors, including the production of antagonistic antibacterial molecules. Bacteroidales species antagonize by both contact-dependent and contact-independent mechanisms. Contact-independent antagonism has been shown to be mediated by antimicrobial toxins that fall into four distinct families: the BSAP (Bacteroidales Secreted Antimicrobial Protein) toxins^1–4^, the peptide bacteroidetocins^5^, the Fab1 toxin^6^, and a ubiquitin-like toxin (BfUbb)^7^. The BSAPs are produced by diverse members of Bacteroidetes and contain membrane attack complex/perforin (MACPF) domains and typically kill strains of the same or similar species, likely by pore formation. Bacteroidetocin peptides are produced by diverse members of the Bacteroidetes and target the essential OM protein BamA^8^ in a wide range of Bacteroidales species. BfUbb and Fab1 are produced by a subset of *B. fragilis* strains and killing is limited to other *B. fragilis* strains.

Most studies of antibacterial toxins in the Bacteroidales have focused on *B. fragilis*. Among the Bacteroidales, *P. vulgatus* (recently reclassified from *Bacteroides vulgatus*^9^) is one of the most ubiquitous and abundant members of the human gut microbiota^10,11^. *P. vulgatus* and *P. dorei* are closely related species^12^ with many commonalities including producing the same O-antigen repeat unit and the production of BSAP-3 by some strains^3^.

Here, we identify and characterize an antibacterial toxin from *P. vulgatus* and *P. dorei* whose primary structure is unrelated to any characterized protein. We show the toxin, designated BcpT, is encoded on a mobile plasmid, requires cleavage at two sites to activate its antibacterial activity, and utilizes the Lipid A-core glycan as its receptor. Exposure of sensitive cells to BcpT induces activation of a sigma/anti-sigma factor pair whose regulon includes genes involved in the synthesis of a protective response to both BcpT and BSAP-3. BcpT defines a previously unidentified family of antibacterial toxins that is widespread in human gut microbiomes.

## Results

### Inhibitory activity of *P. vulgatus* and *P. dorei* strains

**Fig. 1:**
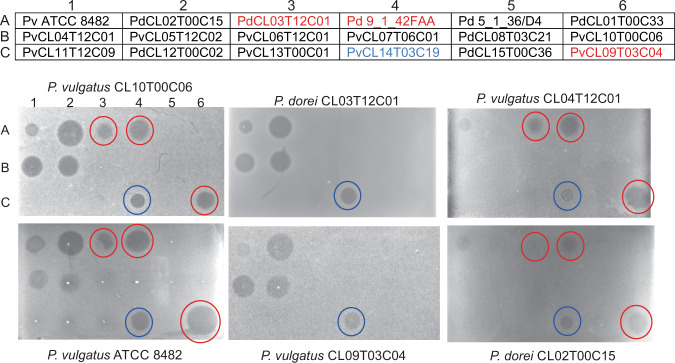
Agar spot overlay assays of *P. vulgatus* and *P. dorei* strains. Upper panel: pattern of the 18 strains (three down, six across) that were spotted onto the plate and grown overnight to test for production of growth inhibitory factor(s) of the overlaid strains. Strains listed in red font produce BSAP-3, the strain listed in blue font produces bacteriodetocin A. Lower panel: The strain names on the top or bottom of the plates indicate the strain that was used as the overlay strain to test for growth inhibition by product(s) secreted by the strains listed above. All overlay assays were performed a minimum of four times with similar results. Source data are provided as a Source Data file.

To analyze the breadth of antimicrobial toxins produced by *P. vulgatus* and *P. dorei* strains, we tested 18 strains (Fig. 1, chart) for production of secreted molecules that inhibit the growth of other *P. vulgatus* and *P. dorei* strains using an agar spot overlay assay^13^. Three of these strains are known to produce BSAP-3 (Fig. 1, spots A3, A4, C6)^3^ and one produces bacteroidetocin A^5^ (Fig. 1, C4). *P. vulgatus* CL10T00C06 (PvCL10) and *P. vulgatus* ATCC 8482 (Pv8482) are known to be sensitive to BSAP-3 and bacteroidetocin A. Here we identified three other strains that produce molecule(s) that inhibit their growth (Fig. 1, left panels) (spots A2, B1, B2), as well as a faint zone of growth inhibition by a molecule produced by Pv8482 (A1). The middle panels of Fig. 1 show sensitivity of two BSAP-3 producing strains, PdCL03T12C01 and PvCL09T03C04 (PvCL09) to molecules produced by this panel of strains. These strains are resistant to their own BSAP-3 toxin as they have an altered receptor^3^. However, both of these strains are sensitive to molecules produced by the strains that targeted PvCL10 and Pv8482 (A2, B1, B2), and are also sensitive to bacteroidetocin A (spot C4). The right-most panels show overlays using PvCL04T12C01 (PvCL04) and *P. dorei* CL02T00C15 (PdCL02) (spots B1, A2), two of the strains shown here to produce an uncharacterized toxin(s). PvCL04 and PdCL02 are relatively resistant to their own toxin(s) and appear to be less sensitive to BSAP-3 and bacteroidetocin A than strain PvCL10.

### Identification of an antibacterial toxin

**Fig. 2:**
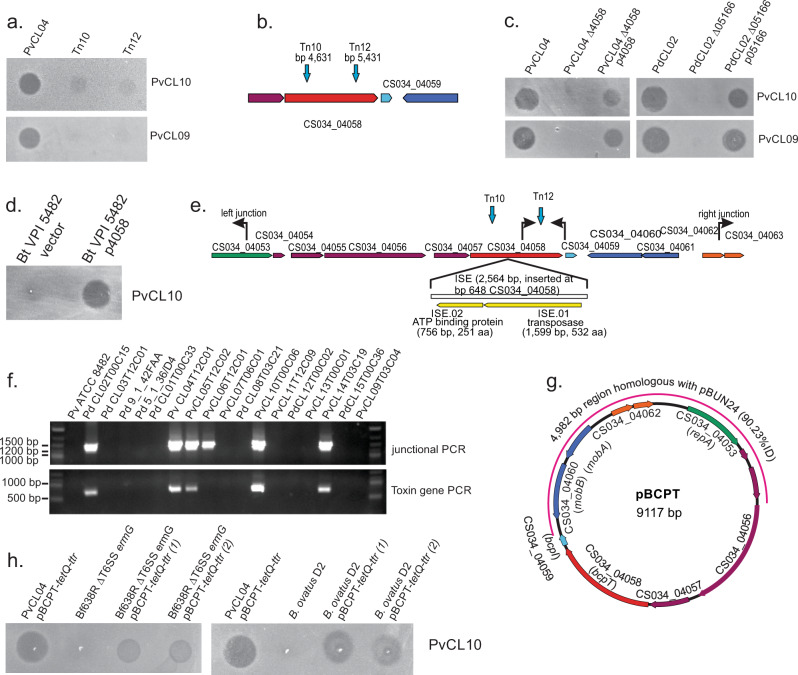
Identification of the toxin-encoding gene on a mobile plasmid. **a** Agar overlay assays of toxin-producing strain PvCL04 and two transposon mutants that abrogate its ability to inhibit the growth of PvCL10 and PvCL09. **b** Genetic context of the gene into which the two transposons inserted. **c** Agar overlay assays showing that deletion of the gene identified by transposon mutagenesis from strains PvCL04 and PdCL02 abrogates toxin activity, which is restored when the gene is added back in trans to these strains. **d** Agar spot overlay assay showing that placement of CS034_04058 in trans in *B. thetaiotaomicron* VPI 5482 confers toxin activity to the strain. **e** Orf map of the contig of the sequenced genome of PvCL04 containing CS034_04058. Primers used to determine if the contig is a circular plasmid and to identify the toxin-producing gene in other strains are shown. Sites of the transposon insertions of mutants Tn10 and Tn12 are shown. An insertion sequence present in a similar contig of strain PvCL10 is shown under the map with its insertion site mapped relative to the contig of PvCL04. **f** EtBr-stained gel showing the results of the PCR using the primers shown in panel E with each of the 18 *P. vulgatus* and *P. dorei* strains listed in Fig. 1a. PCR analyses were performed twice. **g** The circular 9117 pBCPT plasmid of strain PvCL04 showing the portion of the contig that is similar to the Bacteroidales mobile plasmid pBUN24 (pink outer line). The gene shown in green encodes a plasmid replication protein, the genes in blue encode mobilization proteins, and the genes in orange encode a putative toxin-antitoxin system. **h** Overlay assays showing that *B. fragilis* ΔT6SS*ermG* and *B. ovatus* D2 that conjugally received pBCPT-*tetQ-ttr* from PvCL04 pBCPT-*tetQ-ttr* acquire the ability to inhibit the growth of PvCL10. All overlay assays were performed at least twice. Source data are provided as a Source Data file.

The genome sequence of PdCL02 is publicly available (GenBank accession JH724123), and here we sequenced the genomes of PvCL04 and PvCL05T12C02 (PvCL05) to study the genetic basis of toxin production by these strains. We screened a transposon bank of strain PvCL04 for loss of toxin activity and identified two transposon mutants that lost the ability to inhibit the growth of strains PvCL10 and PvCL09 using the agar overlay assay (Fig. 2a). Both transposon insertions interrupted the same gene (CS034_04058) (Fig. 2b). The genomes of PvCL05 and PdCL02 each contain a gene that is identical to CS034_04058, as does the genome of the bacteroidetocin A producing strain PvCL14. CS034_04058 encodes a 499 amino acid protein with a predicted signal peptidase II cleavage site, indicating it is likely an outer surface lipoprotein. Mutants with internal deletions of this gene in both PvCL04 and PdCL02 (the homologous gene is designated HMPREF1063_05166 in PdCL02) do not inhibit growth of either PvCL10 or PvCL09 and toxin activity is restored when the gene is added in trans to these mutants on a plasmid (Fig. 2c). When CS034_04058 is transferred into *B*. *thetaiotaomicron* VPI 5482, it confers toxin activity to this strain (Fig. 2d).

### The toxin is encoded on a small mobile plasmid

The contigs containing the toxin-encoding gene in PvCL04, PvCL05, PdCL02 and PvCL14 are similar in size (9089-9140 bp), suggesting this gene is carried on a plasmid. Using PCR with two outwardly facing primers (Fig. 2e), we confirmed the DNA is circular (Fig. 2f, 2g). We used these primers and a second primer set (Fig. 2e) to PCR amplify the end of the toxin gene, with DNA from all 18 strains tested in Fig. 1 (eight of which do not have available genome sequences). Amplicons across the contig junction are produced for all strains containing the toxin gene. PvCL06T12C01 contained a similar plasmid based on amplification across the junction, but no toxin gene was amplified (Fig. 2f) nor toxin activity detected (Fig. 1). The other unexpected finding is that the toxin gene was amplified using PvCL10 as template (Fig. 2f), however, PvCL10 does not produce this toxin and is very sensitive to it (Fig. 1). Analysis of the PvCL10 genome sequence shows that the toxin gene is interrupted at bp 638 by a 2563 bp insertion sequence disrupting the gene (Fig. 2e).

Further analysis of the sequence of this plasmid revealed that more than half of the plasmid (4982 bp) is 90.23% identical to a previously described ~8.9 kb cryptic plasmid (pBUN24) of *B. uniformis* BUN24^14^, which was isolated from the feces of a 6-year-old girl. Strains of *P. vulgatus*, *Bacteroides intestinalis* and *Parabacteroides distasonis* isolated from the same individual at age 11 also contained plasmids of identical size, two of which are 100% identical to pBUN24, suggesting intra-ecosystem conjugal transfer^14^. The region of pBUN24 that aligns with the plasmid reported here is shown as a pink line of Fig. 2g and includes genes involved in replication, plasmid maintenance and mobilization (Supplementary Table 1,^14^). Four other genes including the toxin gene are not present in pBUN24. Based on this plasmid’s likely mobility via conjugation, we named this toxin BcpT, for **B**acteroidales **c**onjugally transferred **p**lasmid-encoded **t**oxin and the plasmid was named pBCPT. To confirm that pBCPT is mobile, we used strain PvCL04 as the pBCPT donor and first deleted *ermG* from the chromosome, making the strain erythromycin sensitive. Next, we added *tetQ* with a downstream transcriptional termination region (*ttr*) into pBCPT between genes CS034_04061 and 04062 (Supplementary Fig. 1a, b). For transfer studies, this strain was co-cultured on plates with *B. fragilis* 638RΔT6SS*ermG*, which is unable to fire its GA3 T6SS so that it cannot antagonize the donor strain, and *B. ovatus* D2 (naturally erythromycin resistant). Tetracycline and erythromycin resistant transconjugants were selected (Supplementary Fig. 1c, d) and passaged twice and then PCR was performed, which confirmed that pBCPT was transferred to the recipient *B. fragilis* and *B. ovatus* strains (Supplementary Fig. 1e, f). In addition, these two strains were able to inhibit the growth of BcpT sensitive strain PvCL10 in overlay assays, albeit to a lesser extent than the donor PvCL04 (Fig. 2h). These data confirm that pBCPT is a mobile plasmid.

### BcpT activation

**Fig. 3:**
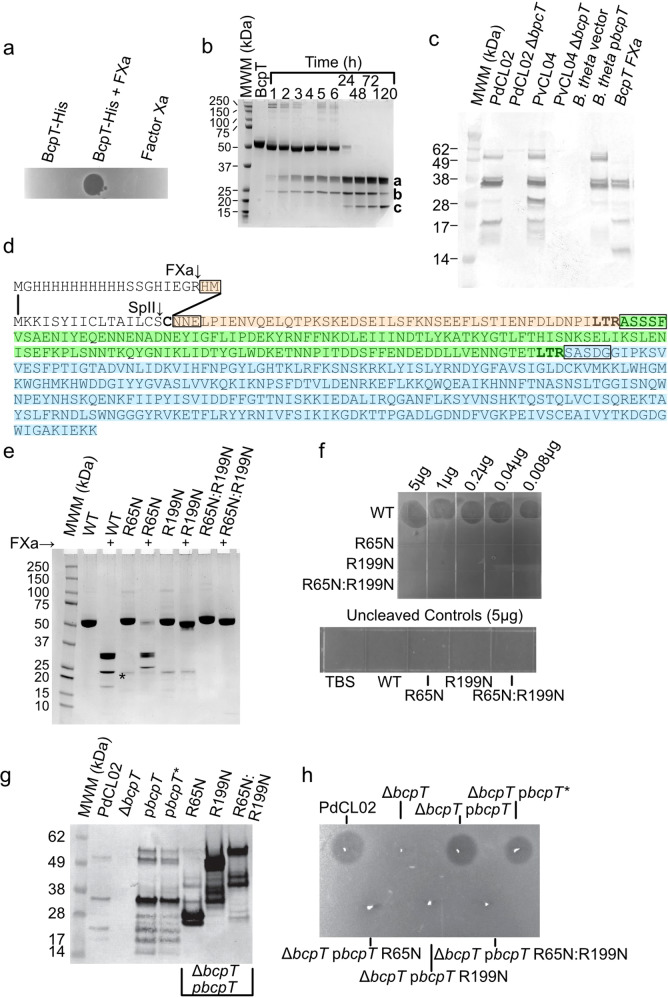
BcpT requires proteolytic cleavage for activity. **a** Agar overlay assays using PvCL10 as the overlay strain with 450 ng His-BcpT, or His-BcpT cleaved with Factor Xa, or Factor Xa alone. **b** Coomassie-stained polyacrylamide gel of His-BcpT cleaved with Factor Xa for various periods of time showing cleavage products a, b, and c. **c** Western immunoblot of supernatants of overnight cultures of the indicated strain or mutant probed with α-BcpT showing a representative of three independent blots. **d** Sequence of BcpT with the native signal sequence showing the SpII cleavage site and the sequence of the construct with the His-tag. The N-terminal sequences of the a, b, and c fragments of panel B determined by amino acid sequencing are boxed. Fragment a is colored blue and fragment c is colored green. Fragment b is the combination of the beige and green sequences. **e** Coomassie-stained gels of recombinant BcpT with mutants at LTR sites, R65N and R199N, cleaved with FXa. The (*) indicates the presence of a probable protein from *E. coli* in that lane and the following three lanes. **f** Agar overlay assays of PvCL10 were spotted with purified BcpT WT and activation site mutants. The horizontal and vertical hashmarks are from the polystyrene plate grids. **g** Western blot of supernatants of overnight cultures of the reconstructed BcpT site mutants R65N, R199N, R65RN199N in the PdCL02 Δ*bcpT* background probed with α-BcpT. **h** Agar overlay assays with spotted  WT PdCL02 and each of the PdCL02 reconstructed BcpT site mutants in the PdCL02 Δ*bcpT* background overlaid with strain PvCL10. The (*) indicates reconstructed plasmid. All overlay assays, Coomassie-stained gels and western blots were performed at least twice. Source data are provided as a Source Data file.

To gain a deeper understanding of the antibacterial activity of BcpT, an N-terminal His-tagged derivative was constructed. The recombinant protein purified from *E. coli* did not exhibit antibacterial activity when spotted onto an overlay of sensitive strain PvCL10 (Fig. 3a). Predicting that the N-terminal His-tag may inhibit activity, we used Factor Xa (FXa) to remove the His-tag. This resulted in antibacterial activity (Fig. 3a), suggesting that the tag inhibited toxin activity. However, analysis of the cleavage pattern produced when the His-tagged BcpT is treated with FXa revealed it cleaves not only at the FXa specific site (I-E-G-R), but also at other sites, resulting in three fragments, not including the His-tag (Fig. 3b). Fragments a and b appear first, whereas fragment c appears upon longer incubation times and coincides with a decreased abundance of fragment b. N-terminal sequencing showed FXa cleaves after R65 and R199 of two Leu-Thr-Arg (LTR) motifs (Fig. 3d). Fragment a encompasses residues 200–499, fragment c encompasses residues 66-199, and fragment b encompasses residues 18-199. Fragment b is an intermediate cleavage product when the R65 site is uncleaved, as FXa cleaves more slowly at R65. Culture supernatants of PdCL02 and PvCL04 revealed BcpT fragments similar in size to those generated by FXa cleaved recombinant BcpT (Fig. 3c) demonstrating that BcpT cleavage occurs in the producing strains. BcpT is also present in cleaved form in the supernatant of *B. thetaiotaomicron* VPI-5482 when *bcpT* is provided in trans (Fig. 3c), demonstrating that this protease activity is not specific to toxin-producing strains. BcpT has two potential sites of glycosylation^15^, and therefore the migration of these fragments may be slightly altered compared to those generated from the recombinant protein.

To test whether cleavage at R65 and/or R199 is required for BcpT activation, single and double asparagine substitutions of R65 and R199 were generated, the proteins were purified and treated with FXa. BcpT^R65N^ is cleaved only at the R199 site, whereas BcpT^R199N^ and BcpT^R65N:R199N^ are not cleaved by FXa (other than releasing the His-tag) (Fig. 3e). Agar spot overlay assays using purified FXa treated His-BcpT site mutants showed that these altered BcpT proteins fail to inhibit the growth of PvCL10 (Fig. 3f), indicating cleavage at one or both sites is necessary for activity (Fig. 3f). This difference in activity was not due to aberrant folding as the chymotrypsin digestion profiles of all three mutant proteins are similar to WT at various ratios of chymotrypsin to protein (Fig. S2).

To confirm that R65 and R199 are necessary for cleavage of native BcpT in the producing strain, BcpT site mutant constructs were altered to replace the His-tag with the native signal sequence, cloned into a Bacteroidales expression vector, and transferred into PdCL02 Δ*bcpT*. The reconstructed wild-type construct, designated pbcpT*, is cleaved in the same manner as the original p*bcpT* construct, whereas the Asn site mutants at R65 or R199 result in aberrant cleavage patterns (Fig. 3g) and are inactive (Fig. 3h).

### Identification of proteases that activate BcpT

The rate of BcpT cleavage by FXa is slow, typically taking 24–48 h to fully cleave BcpT and this cleavage was serendipitous, as the LTR sequence is not a known recognition site for FXa. Fragipain (Fpn) is a C11 protease family member and is required for activation of *B. fragilis* enterotoxin, Bft^16^, which affects epithelial cells^17^. Fragipain also releases many proteins from the surface of *B. fragilis*^18^. Fpn-like proteases are widely distributed in the Bacteroidetes phylum^18^. Fpn cleaves Bft at a QTR site and based on the activation sites of BcpT (LTR), we predicted an Fpn-like protease(s) cleaves and activates BcpT. Two genes of the PdCL02 genome, HMPREF1063_01439 and HMPREF1063_00451, encode proteins that are 48% and 45% similar to Fpn, respectively, and 47% similar to each other. We designated these proteins doripain A (DpnA) and doripain B (DpnB), which are conserved in *P. dorei* and *P. vulgatus* strains. Both DpnA and DpnB contain SpII cleavage sites with carboxylate containing residues just after the lipidated cysteine suggesting they are transported to the surface^19^, as has been shown for Fpn^18^.

**Fig. 4:**
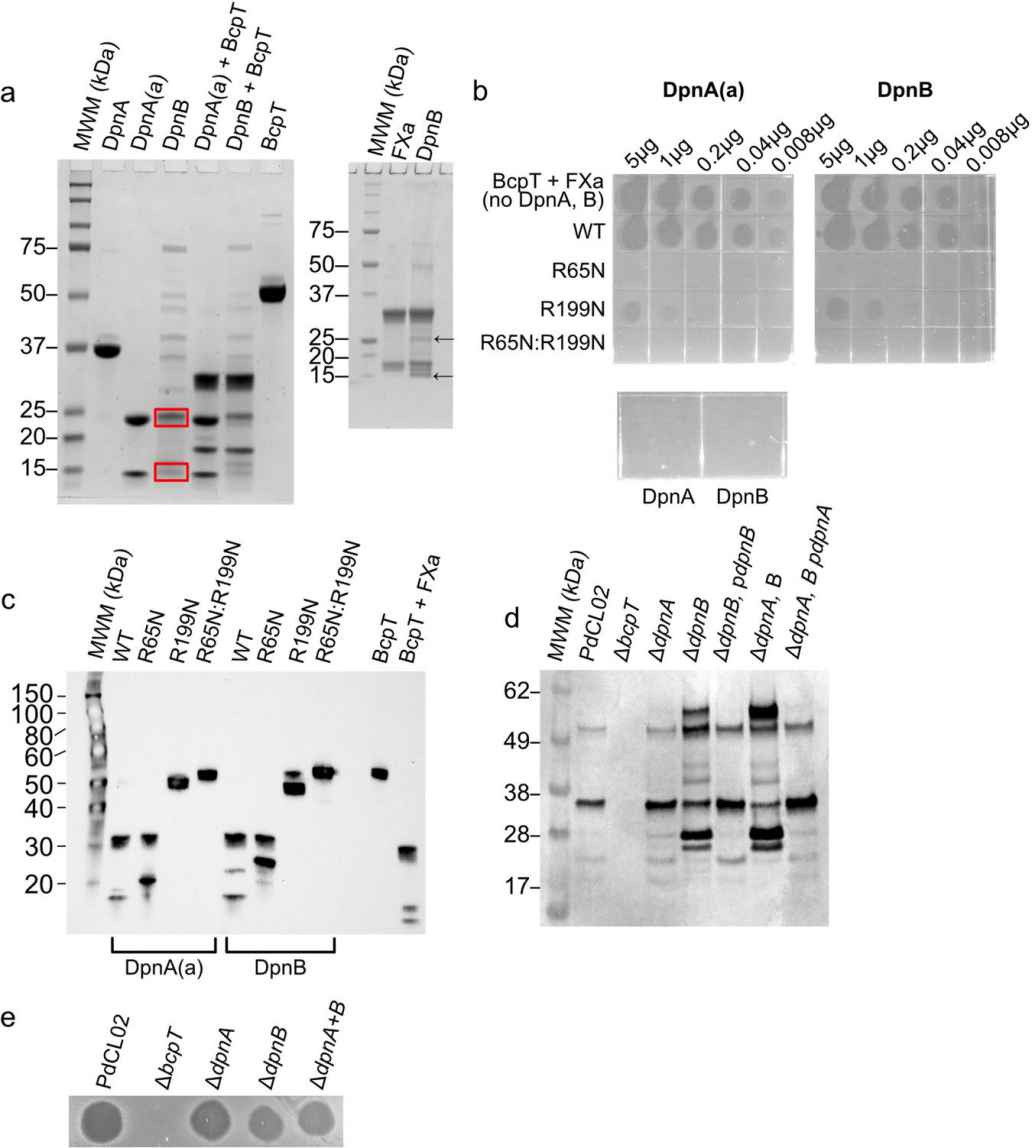
C11 family proteases of *P. dorei* activate BcpT. **a** Left panel: Coomassie-stained gel of purified recombinant doripains (Dpns) and their ability to cleave BcpT. Purified recombinant doripain A (DpnA) incubated with trypsin at a 1:50 (w:w) trypsin to DpnA ratio for 15 mins at 37 °C was sufficient to cleave the activation loop without degradation of the protein. DpnA activated in this manner is referred to as DpnA(a). Red boxes indicate cleaved doripain B (DpnB) fragments that eluted with contaminating proteins from *E. coli*, possibly due to their association with DpnB. Right panel: FXa and DpnB cleaved BcpT showing the same sized fragments are generated. Arrows indicate the DpnB protease fragments that result from its activation **b** Agar overlay assays showing that the Dpns can activate BcpT like FXa and inhibit the growth of Pv8482. Dpns did not activate the R65N or the R199N-R65N double mutants, however, a weak activation of the R199N was observed. Although this assay is only semi-quantitiative, the spot of the undiluted R199N (5 μg) most closely resembled that of the 0.04 μg spot of the WT, which is <10% of WT activity. **c** Western immunoblot of purified BcpT and site mutants of BcpT cleaved by DpnA(a) and DpnB. The blot was probed with α-BcpT. Note that the smaller fragment in the R65N in the DpnA is smaller than that of the DpnB cleaved R65N mutant. We have found that DpnA cleaves the His-tag more efficiently than DpnB which accounts for the difference in size of the small fragment of ~2.5 kDa. **d** Western immunoblot analysis of BcpT cleavage products in the supernatant of PdCL02 and *dpn* mutants and complemented mutants. **e** Agar overlay assay analyzing the ability of PdCL02 *dpn* mutants to inhibit the growth of PvCL10. Overlays and BcpT cleavage analyses were performed two to three times with consistent results. All gels and blots were perfomred a minimum of two times with consistent results. Source data are provided as a Source Data file.

The *dpnA* and *dpnB* genes were codon optimized for expression in *E. coli* and cloned into pET21b removing the signal sequence and creating an N-terminal His-tagged derivative. Fpn, like most C11 proteases, contains an activation loop that occludes the active site of the protein prior to being cleaved^20^. Like Fpn, recombinant DpnB is cleaved during expression and purification from *E. coli*, producing two fragments (Fig. 4a). In contrast, DpnA remains full-length and cannot activate BcpT (Fig. 4a) unless treated with trypsin to cleave its activation loop, hereafter referred to as DpnA(a) (Fig. 4a, b). The active forms of both proteases cleave and activate BcpT (Fig. 4a, b). Furthermore, both proteases can cleave BcpT^R199N^ at the R65 site, which FXa does not (Fig. 4c). BcpT^R65N^ cleaved with DpnA(a) or DpnB exhibits no activity and BcpT^R199N^ cleaved with these proteases only possesses slight activity (<1% WT) (Fig. 4b), therefore, full activity requires cleavage at both LTR sites.

To determine the role of DpnA and/or DpnB to the cleavage of BcpT in vivo, we deleted both *dpn* genes individually and together in PdCL02 and analyzed BcpT fragments in the supernatants and BcpT activity in agar overlays. There are no differences in the sizes of the BcpT fragments found in the supernatant of PdCL02 *∆dpnA* versus WT (Fig. 4d), but the PdCL02 Δ*dpnB* mutant has an altered cleavage pattern with more of the toxin in the uncleaved form. The WT cleavage pattern is restored when *dpnB* is restored in trans to PdCL02 Δ*dpnB*. Deletion of both *dpn* genes reveals a BcpT cleavage pattern like that of Δ*dpnB* (Fig. 4d), however, enough of the activated toxin is still present to inhibit the growth of sensitive strain PvCL10 (Fig. 4e). Together, these data indicate that BcpT can be cleaved by both doripains in vitro although DpnB appears to be primarily responsible for directly or indirectly cleaving BcpT in the bacteria, and partial cleavage of BcpT occurs in the absence of both proteases.

### Identification of the toxin receptor

To identify the BcpT receptor, transposon banks were generated in PvCL10 and enriched for resistant mutants by co-culture with the *bcpT*^+^ strain PdCL02. The surviving transposon mutants were individually screened in overlay assays. Several transposon mutants partially resistant to the toxin were identified (discussed below), however, no mutants that were completely resistant were selected in this screen, suggesting that BcpT interacts with an essential molecule.

**Fig. 5:**
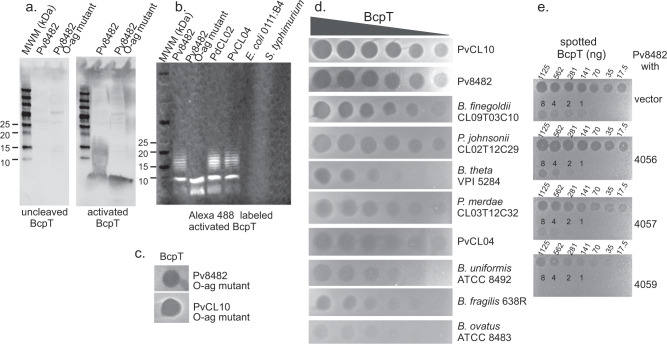
Identification of BcpT receptor. **a** Blots of membranes of Pv8482 WT and O-antigen mutant probed with unactivated or activated BcpT and detected with affinity-purified α-BcpT. **b** Blot of purified LPS from the indicated strains probed with Alexa 488 labeled activated BcpT. **c** Overlay assays of O-antigen mutants of PvCL10 and Bv8482 to purified BcpT. **d** Agar overlay assays showing the sensitivity of various Bacteroidales species and strains to BcpT. The left most spot of each overlay is 1 µg of toxin and each subsequent spot is a two-fold dilution. **e** Agar spot overlay assays showing the ability of each of three pBCPT genes to protect PvCL10 from BcpT toxicity when expressed in trans. Overlay assays were performed twice and receptor probing blots were performed twice with consistent results. Source data are provided as a Source Data file.

As a genetic approach failed to reveal the receptor of BcpT, we used a receptor blot technique^21^. We probed a Western blot containing purified membranes from the sensitive Pv8482 strain and its cognate O-antigen mutant with proteolytically activated and unactivated BcpT. Binding of BcpT to membrane components was detected using affinity-purified rabbit α-BcpT. Activated BcpT binds a low molecular weight molecule in the O-antigen mutant that is the size of the Lipid A-core glycan^3^, and BcpT binds larger and heterogeneously sized molecules in the WT, indicative of a laddering O-antigen (Fig. 5a). Unactivated BcpT and the cleaved R65N, R199N and R65N/R199N site mutants do not exhibit any detectable binding (Fig. 5a, Supplementary Fig. 3a). These data strongly suggest the receptor is the Lipid A-core glycan of the LPS and that unactivated BcpT is unable to bind. As confirmation that BcpT binds the Lipid A-core glycan, LPS and the Lipid A-core were purified from Bv8482 WT and the Bv8482 O-antigen mutant, respectively, as well as LPS from the BcpT-producing PvCL04 and PdCL02 strains (Supplementary Fig. 3b). Blots containing these molecules were probed with BcpT labeled with the sulhydryl-specific maleimide derivative of Alexa Fluor 488, which labels one or more of the 3 cysteine residues in BcpT (C270, C412 and C479) but does not affect its antibacterial activity (Supplementary Fig. 3d). These analyses confirmed that activated BcpT binds the Lipid A-core glycan portion of the LPS molecule of *P. vulgatus* and *P. dorei* strains but not the LPS of *E. coli* or *Salmonella typhimurium* (Fig. 5b, Supplementary Fig. 3c), which are not growth inhibited by activated BcpT (Supplementary Fig. 3e). In addition, BcpT is potent against O-antigen mutants of both Pv8484 and PvCL10 (Fig. 5c). Mutations that affect portions of the core glycan are lethal in many Gram-negative bacteria, likely explaining why the genetic approach failed to identify the receptor,

To better understand the fragments of BcpT that are involved in receptor binding, we performed receptor blocking assays. We found that purified recombinant amino terminal fragments 18-199 and 65-199 effectively block binding of activated BcpT to the Lipid A-core. When each fragment was mixed at a 10-molar excess to activated, purified BcpT in the blot probe buffer, incubated overnight, washed and then probed with anti-BcpT, binding of activated BcpT to the Lipid A-core was eliminated (Supplementary Fig. S4). Furthermore, these blots show that neither amino terminal fragment binds to the Lipid A-core since the antibody recognizes both these fragments (Fig. 3c, g). Hence, these data show that the C-terminal 200–499 fragment binds the receptor and that the N-terminal fragment likely blocks this binding site. These data also strongly suggest that the antibacterial activity is contained within the 200–499 fragment. We were unable to purify the 200–499 fragment to test its binding in the absence of the 18-199 fragment, which suggests that the amino terminal fragment may be important to the folding and/or stability of the 200–499 fragment.

As related species often share a common Lipid A-core glycan, we tested the ability of purified, recombinant BcpT to inhibit the growth of seven additional *Bacteroides*, *Phocaeicola* and *Parabacteroides* species (Fig. 5d). BcpT sensitivity varied wherein some strains, such as *B. finegoldii* CL09T03C10 and *P. johnsonii* CL02T12C29 are inhibited at low BcpT concentrations. In contrast, *B. fragilis* 638R and *B. thetaiotaomicron* VPI-5482, are only affected by the highest BcpT concentrations and *B. ovatus* ATCC 8483 is the most resistant. Although the structure of the core glycan of a *P. vulgatus* strain has been elucidated^22^, the structure of the core glycan has not been determined for other strains or species, which will be necessary to determine whether the basis for the varying sensitivities is based on the structural differences. Purified BcpT also binds the LPS of the BcpT-producing strains PvCL02 and PvCL04 (Fig. 5b), yet PvCL04 is less sensitive to purified recombinant BcpT than PvCL10 and Bv8482. In addition, native BcpT produced from these strains does not potently inhibit them in overlay assays (Fig. 1), suggesting the presence of an immunity protein.

### Identification of a BcpT immunity protein

Immunity proteins are typically encoded just downstream of the toxin gene. Of the open reading frames of the pBCPT plasmid, only three do not have obvious functions in other cellular processes (CS034_4056, CS034_4057, and CS034_4059) (Fig. 2g). Strain PvCL10 also contains the pBCPT plasmid, with a 2564-bp insertion in *bcpT* that inactivates it (Fig. 2e) and this strain is highly sensitive to BcpT (Fig. 1). As genes CS034_4056 and CS034_4057 are upstream of the gene disruption, their expression should not be altered in strain PvCL10, however, the small gene downstream of *bcpT* may be transcriptionally affected by the insertion, making this the most likely immunity gene candidate. Each of these three genes was cloned into expression plasmid pFD340 and placed in trans in Pv8482. Overlay assays were performed with dilutions of activated BcpT, revealing that the small 174 bp gene CS034_4059 just downstream of *bcpT* confers at least 8-fold protection to BcpT, whereas genes CS034_4056 and CS034_4057 confer no protection (Fig. 5e). Gene CS034_4059 encodes a protein of 57 aa (named herein BcpI), which has an SpII signal sequence suggesting it is a lipoprotein tethered to the outer membrane. BcpI lacks carboxylate containing residues just after the lipidated cysteine that suggest it is flipped to the outer surface^19^ and therefore, is likely on the periplasmic side.

### BcpT induces a protective stress response

**Fig. 6:**
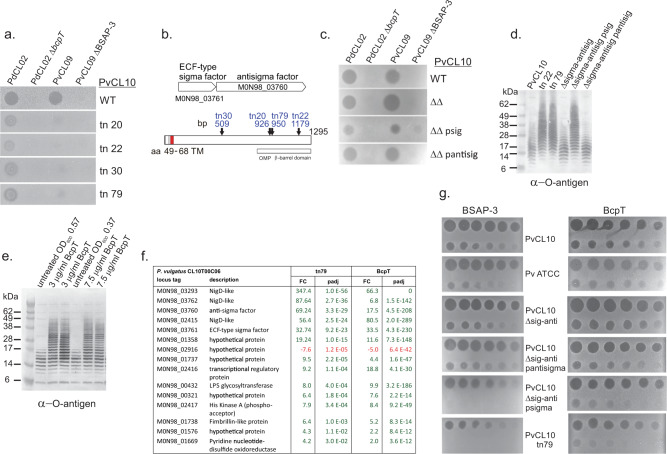
A BcpT-induced protective regulon. **a** Agar spot overlay assays of four transposon (tn) mutants of PvCL10 that were slightly protected from BcpT and substantial protected from BSAP-3. **b** Orf map of the anti-sigma factor encoding gene (M098_03760) into which all transposons inserted showing the genetic context of the upstream gene encoding the cognate sigma factor (M0N98_03761). Bottom panel shows the regions of the anti-sigma factor including the inner transmembrane region shown in red and the outer membrane β-barrel region and sites of each of the four transposon mutants. **c** Agar spot overlay assays showing the sensitivity of the mutant strains of PvCL10 (indicated on right) to each of the toxin-producing strains or the toxin deletion mutants. **d** Western immunoblot showing LPS O-antigen sizes of various PvCL10 WT and mutant strains (psig and pantisig are plasmids containing the respective gene). The blot was probed with antiserum to PvCL10 O-antigen. **e** Western immunoblot of whole cell lysates of PvCL10 treated with two concentrations of BcpT for 3 h in duplicate or OD_600_ matched untreated controls. Bacterial equivalent to 4 µl of the culture were added to each well. The blot is probed with antiserum to the PvCL10 O-antigen. **f** List of the 15 genes that are differentially expressed using the strict cut-off criteria listed in the methods section in PvCL10 tn79 compared to WT PvCL10. Green indicates upregulation and red indicates downregulation. FC represents the fold-change and padj represents the *p*-value adjusted for multiple comparisons by DESeq2. All 15 genes are also differentially expressed when PvCL10 is treated with BcpT. **g** Agar spot overlay assays showing the susceptibility of various strains to dilutions of BSAP-3 and BcpT. The first spot of the BSAP-3 plates is 1 µg and the first spot of the BcpT plates is 3 µg and each consecutive spot is a 2-fold dilution left to right on the top row and then left to right on the bottom row. Overlay assays and western immunoblots were performed at least twice with consistent results. Source data are provided as a Source Data file.

As indicated above, the PvCL10 transposon mutant screen revealed four mutants partially resistant to BcpT (Fig. 6a). Unexpectedly, these mutants exhibited an increased resistance to BSAP-3 (Fig. 6a), the MACPF toxin produced by PvCL09, which uses the O-antigen of sensitive *P. vulgatus* and *P. dorei* strains as its receptor^3^. These transposon insertions all mapped to M0N98_03760, a gene encoding an unusual anti-sigma factor (Fig. 6b), which is downstream of a sigma factor gene (M0N98_03761). Most anti-sigma factors have cytoplasmic, transmembrane and periplasmic domains. The cytoplasmic domain sequesters the sigma factor until an extracytoplasmic signal is detected releasing the sigma factor to initiate transcription of its regulon (reviewed in^23^). The anti-sigma factor encoded by M0N98_03760 is much larger and is predicted to span the periplasmic space with a β-barrel outer membrane domain at the C-terminus (Fig. 6b). A similar ECF-sigma factor/anti-sigma factor pair was described in *B. fragilis* and shown to respond to oxidative stress^24^. Full-length orthologs of this gene pair are present in all *Bacteroides/Phocaeicola* species analyzed, although anti-sigma factor similarity is low between species (~50–70% similarity). Among *P. dorei* and *P. vulgatus* strains, these proteins are >98% (sigma factor) and 95% (anti-sigma factor) identical. Attempts to delete the anti-sigma factor gene from PvCL10 were not successful, but we could simultaneously delete both the sigma factor and anti-sigma factor genes and then restore each gene individually in trans. The partial protection from BcpT and BSAP-3 toxicity was reproduced in strains with an active sigma factor (i.e. no functional anti-sigma factor gene), but not in strains where the anti-sigma factor is intact or when both genes are deleted (Fig. 6c). Hence, these results show that the regulon of this sigma/anti-sigma pair likely includes factors that provide partial protection to both BcpT and BSAP-3.

The receptor of BSAP-3 is the O-antigen; however, as determined above, it is not the receptor of BcpT (Fig. 5c). Strains in which the anti-sigma factor is impaired (allowing the sigma factor to transcribe its regulon) synthesize LPS molecules with substantially more O-antigen repeat units than the sensitive WT strain (Fig. 6d). These data likely explain why these mutant strains are protected from BSAP-3: longer O-antigens would place BSAP-3 too far from the membrane to insert its β-barrel pore. It is possible that longer O-antigen chains may impair BcpT access to the Lipid A-core. As the induction of the sigma/anti-sigma factor regulon provided partial protection to BcpT, we sought to determine whether BcpT exposure induces this protective response.

We first treated sensitive strain PvCL10 with 3 µg/ml or 7.5 µg/ml BcpT for 3 h, which resulted in much longer O-antigens compared to untreated bacteria (Fig. 6e). These data suggest that BcpT induces the protective sigma factor regulon. Transcriptomic analyses were performed on untreated and BcpT-treated PvCL10 where BcpT was added at a concentration that allowed for slow, rather than arrested growth. We also performed transcriptomic analysis of PvCL10 transposon mutant 79, a mutant where the anti-sigma factor gene was interrupted, which confers partial protection to BcpT (Fig. 6a). After 3 h of BcpT treatment, the M0N98_03761 sigma factor gene was upregulated 33-fold (adjusted *p*-value of 4e-230) (Supplementary Data 1, Fig. 6f). In PvCL10 transposon mutant 79, there were only 15 differentially expressed genes (DEGs) by the strict cut-off  used, 14 of which are upregulated compared to WT PvCL10. Importantly, all 15 of these genes are also differentially regulated in the same manner in BcpT-treated cells (Supplementary Data 1, Fig. 6f). BcpT treatment also induced numerous other genes, many of which were shown to be induced upon treatment with bacteroidetocin A (Supplementary Data 1), which binds and inhibits BamA leading to severe outer membrane defects and cell death^8^. These data suggest that BcpT-treated bacteria are responding to cell envelope stress, which is likely the signal for induction of the sigma/anti-sigma regulon.

To better quantify the protective effect of the M0N98_03760-61 regulon, we analyzed the degree to which the various PvCL10 sigma/anti-sigma factor mutant strains are protected when treated with BSAP-3 or BcpT. We show that the mutants in which the anti-sigma factor is interrupted (PvCL10 tn mutant 79) or missing (delta sigma/antisigma psig) survive exposure to a 100-fold higher concentration of BSAP-3 and ~8-fold higher concentration of BcpT compared to WT (Fig. 6g).

### Prevalence of *bcpT* in genomes and human gut metagenomes

The prevalence of *bcpT* and pBCPT was examined among 1148 non-redundant Bacteroidales genomes with species designations, including strains from 14 different families and 41 different genera (Supplementary Data 2). We used blastn to search for the 1500-bp *bcpT* gene and for the 5058-bp plasmid backbone, which excludes the four genes specific to pBCPT. *bcpT* was present (98.8 – 100% identity) only in genomes of *P. dorei* (4/26 strains) and *P. vulgatus* (10/88 strains) with one exception, *Parabacteroides goldsteinii* 910340 isolated from a blood sample in Denmark^25^. In most strains, the *bcpT* ortholog was contained on a contig ranging from 9.1 – 9.5 kb that matched the pBCPT backbone at 99-100% DNA identity. *B. ovatus* An161, isolated from the cecum of a chicken in China^26^, was the only other *Bacteroides* genome that encoded a product similar to BcpT. The ortholog from this strain (B5F02_25665) is 78.4% identical to *bcpT* and is contained on a 9079 bp contig, but it does not align with pBCPT (Supplementary Data 2). Using blastp, we found BcpT orthologs with less similarity (~50–70% similar) encoded in three *Prevotella intermedia*, two *Prevotella enoeca* and one *Prevotella shahii* strains (Supplementary Data 2). These orthologs are present on large contigs, indicating they are likely not plasmid encoded. Although pBCPT is primarily restricted to *P. dorei* and *P. vulgatus*, plasmids that aligned with the backbone of pBCPT (lacking *bcpT*) at lower identity were identified in 15 different Bacteroidales species (Supplementary Data 2).

To determine the prevalence of *bcpT* in sequenced human gut metagenomes and to determine if there are geographical differences, we analyzed 15 different human gut metagenomics datasets including 1767 non-longitudinal metagenomes from different geographies (Supplementary Data 3). These data show that the Japanese dataset has the highest prevalence of metagenomes with *bcpT* (20.7% positive), followed by a US dataset (18.5% positive). Gut metagenomics datasets from Madagascar (112 metagenomes), Ethiopia (24 adult metagenomes) and Peru (36 metagenomes), collectively have a single metagenome with *bcpT*. This observation is consistent with a recent study showing a higher rate of horizontal gene transfer in more industrialized populations^27^. Of the 1767 human gut metagenomes analyzed, 225 contain *bcpT* based on our inclusion criteria. Of these 225 *bcpT*-positive metagenomes, analysis using MetaPhlAn^28^ indicated that all but two of these metagenomes have either *P. vulgatus* or *P. dorei* present at a detectable level (Supplementary Data 3). In total, these genomic and metagenomics data indicate that pBCPT is present in numerous countries, yet absent from certain human populations, with a strong bias for *P. vulgatus* and *P. dorei* strains.

## Discussion

BcpT is the first antibacterial toxin of the gut Bacteroidales shown to be plasmid-encoded. We show that pBCPT is mobile and can be transferred to other Bacteroidales species, and therefore, the basis for its rather restricted presence in *P. vulgatus* and *P. dorei* strains remains an enigma. Although we show that BcpT can inhibit the growth of other Bacteroidales species, presumably due to similarity of their Lipid A-core glycan structure, it is possible that BcpT is most beneficial to *P. vulgatus* and *P. dorei* when competing in the gut, and therefore, there may be different benefit-cost ratios in Bacteroidales species. The toxin exhibits unusual features, including the necessity for two proteolytic cleavage events for receptor binding and activity. As far as we know, the dual cleavage required to activate BcpT has not been observed for other characterized proteolytically activated toxin, which require only a single cleavage for activation (e.g., anthrax toxin, diphtheria toxin^29^ Bft, bacteroidetocins, Fab1^6^, class IIa bacteriocins, etc) and is the first antimicrobial toxin produced by the gut Bacteroidales shown to bind the Lipid A-core glycan. It is yet unknown if BcpT is glycosylated and if so, how such glycosylation would affect its processing, activity, and stability.

The Lipid A-core receptor of BcpT, the likely location of the immunity protein in the OM and the upregulation of a  cell  envelope stress-associated regulon suggests BcpT perturbs the OM. The interaction of activated BcpT with the Lipid A-core is blocked by its amino terminal domain (residues 18-199), which itself does not bind the Lipid A-core. This suggests that proteolytic activation allows the Lipid A-core to displace the amino terminal fragment and access the BcpT receptor binding site. Both proteolytic cleavages are likely necessary to allow the receptor to displace the amino terminal fragment and access the binding, as the proteolytically treated R65N and R65N-R199N mutants lacked any activity and the R199N exhibited <10% of the activated WT BcpT. The fact that we could not purify the C-terminal domain (residues 200–499) also suggests that the amino terminal domain acts as an intramolecular chaperone sequence that is also important for the stability and/or folding of the C-terminal fragment. These data also strongly suggest that it is the C-terminal fragment (residues 200–499) that exerts the antibacterial activity. The operon containing BcpT also contains a small lipoprotein, BcpI, that acts as an immunity protein. As BcpT can bind its host cell’s lipid A-core, an immunity protein is necessary to protect the producing cell. The antibacterial mechanism of BcpT remains unknown, and it does not share any primary structure similarity with a characterized toxin that would allow us to speculate on its mechanism.

BcpT exposure induces a regulon controlled by the unusual sigma/anti-sigma factor pair and correlates with a partial protection of cells to BcpT and BSAP-3. This protection may be multi-genic with different phenotypes conferring protection to different toxins; e.g, the upregulation of the glycosyltransferase gene of the LPS glycan region may account for protection to BSAP-3 that uses O-antigen as its receptor, whereas protection to BcpT may be mediated by different factors of this regulon. Interestingly, genes encoding three NigD orthologs are highly upregulated by BcpT treatment. NigD is a putative lipoprotein encoded in the nigrescin locus of *Prevotella nigrescens*. NigD was suggested to be an immunity protein for nigrescin (NigC)^30^, which was shown to have antibacterial activity against *Porphyromonas gingivalis*^31^ but its mechanism remains unknown. Understanding this unusual sigma/anti-sigma factor pair and its regulon will be paramount to understand how *Bacteroidales* respond to various stresses, especially as relates to bacterial and/or host produced antibacterial molecules.

The combined data suggest that the effects of BcpT on sensitive cells is likely complex and conditionally dependent, ranging from toxic to protective.

## Methods

All primers used in this study are listed in Supplementary Table 2.

### Bacterial strains and growth conditions

Bacterial stains used in this study are listed in Supplementary Table 3. Bacteroidales strains were grown in basal liquid medium^32^ or on BHIS plates^33^. Antibiotics used for selection include erythromycin (10 µg/ml), gentamicin (200 µg/ml), tetracycline (4 µg/ml) carbenicillin (100 µg/ml), and kanamycin (50 µg/ml).

### Agar spot overlay assay

 Bacterial strains were dotted in 5 µl aliquots from broth cultures onto rectangular BHIS plates and grown overnight. Bacteria were removed using two applications of filter paper and plates were exposed to chloroform vapor for 15 min to kill residual bacteria^13^. Strains being tested for sensitivity were grown in basal medium to an OD_600_ of ~ 0.7 and added to 6 ml top agar (BHIS 0.75% agar), which was the spread over the plate and grown anaerobically overnight. For toxin spotting assays using recombinant BcpT or BSAP-3, strains were grown overnight, added to top agar and poured onto BHIS plates. After hardening, dilutions of BcpT samples (5–7.5 μl) were spotted unto the top agar. Plates were incubated under anaerobic conditions at 37°C overnight.

### Transposon mutagenesis of PvCL04 and PvCL10

Random mutagenesis of PvCL04 was performed using the transposon containing plasmid pYT646b and individual mutants were screened using the agar spot overlay assay for those that no longer inhibited the growth of PvCL10. The insertion sites of tn10 and tn12 of PvCL04 were identified by a  previously described method^34^. Random mutagenesis of PvCL10 was performed using pSAM_BcellWH2 and the insertion sites identified by PCR as described^35^.

### Creation of deletion mutants and complementing clones

An internal non-polar deletion mutant of *bcpT* of PdCL02 (HMPREF1063_05166) was constructed by amplifying DNA upstream and downstream of the gene using the primers listed in Supplementary Table 2. PCR products were cloned by three-way ligation into pBluescript utilizing blue and white selection, and then excised using BamHI and MluI and cloned into pKnock-*bla-ermGb*. This plasmid was conjugally transferred from *E. coli* into PdCL02. Cointegrates were selected by gentamycin/erythromycin resistance. Double cross-outs were screened via PCR for mutant genotype.

Deletion of *bcpT* of PvCL04 (CS034_04058) was constructed by amplifying DNA upstream and downstream of the region to be deleted and cloning by three-way ligation into BamHI/MluI digested pBluescript utilizing blue and white selection. This construct was then transferred into pKnock-*bla*-*tetQ* and conjugally transferred from *E. coli* to PvCL04. Cointegrates were selected by gentamycin/tetracycline resistance. Double cross-out recombinants were screened via PCR for mutant genotype.

Deletion of genes *dpnA* (HMPREF1064_01439) and *dpnB* (HMPREF1064_00451) from PdCL02T12C06 was created by amplifying regions upstream and downstream of the genes and cloning using NEBuilder into BamHI-digested pLGB13^33^. The construct was transferred from *E.col* into PdCL02. Cointegrates were selected on gentamycin/erythromycin and double recombination cross-outs were selected on BHIS with anhydrotetracycline (aTC, 50 ng/ml) and screened via PCR for mutant genotype.

The internal, non-polar deletion mutant of the PvCL10 sigma factor/anti-sigma factor genes (M0N98_03760-61) was constructed by amplifying DNA upstream and downstream of the region to be deleted and cloned into the BamHI site of pKnock-*bla*-*ermGb* using NEBuilder, transformed into *E. coli* S17 λ pir, and conjugally transferred into PvCL10. Cointegrates were selected on gentamycin/erythromycin. Double cross-out recombinants were screened via PCR for mutant genotype.

Genes expressed in trans, including those used to complement the mutants described above, were PCR amplified and ligated into BamHI-digested pFD340^36^ and conjugally transferred from *E. coli* to Bacteroidales.

### Identification of immunity gene

The three genes unique to pBCPT that are not contained into pBUN24 (CS034_04056, CS034_04057, and CS034_04059) (Fig. 2g) were cloned individually into expression vector pFD340 using NEBuilder. The resulting plasmids were conjugally transferred into Pv8482 and the ability of the cloned product to protect Pv8482 from growth inhibition by activated BcpT was quantified in overlay assays compared to Pv8482 with vector alone.

### pBCPT mobilization assays

To determine if pBCPT could be mobilized to other Bacteroidales species, we started by making the donor strain (PvCL04) erythromycin sensitive and added *tetQ* to pBCPT. First, *ermG* was deleted from PvCL04 by amplifying regions upstream and downstream of ermG (CS034_04137) and cloning them into BamHI-digested pLGB30^33^ using NEBuilder. Cointegrates were selected on tetracycline/gentamycin and double cross-outs were selected by plating on BHIS with 10 mM rhamnose. Next, *tetQ* was amplified with its own promoter from strain *B. caccae* CL03T12C61. The strong transcriptional termination region (*ttr*) downstream of BF638R_1994 was PCR amplified for placement downstream of *tetQ* to prevent transcriptional readthrough into other pBCPT genes. The *tetQ*-*ttr*was inserted between the two pBCPT divergently transcribed genes CS034_04061-62 in a manner so as not to affect the promoters of either gene (Supplementary Fig. 1b). The DNA regions flanking each side of this insertion site were PCR amplified and the four PCR products (left flank, *tetQ*, *ttr*, right flank) were cloned directionally into BamHI-digested pLGB13. The correct assembly of these pieces was confirmed by whole plasmid sequencing and the plasmid was conjugally transferred into PvCL04 Δ*ermG* and cointegrates were selected on erythromycin/gentamycin. Double cross-outs were selected using BHIS plates containing aTC.

For recipient strains, we used *B. ovatus* D2, which is ErmR and TetS, and constructed an erythromycin resistant strain of *B. fragilis* 638RΔT6SS^37^, which lacks the ability to kill by the GA3 T6SS. *ermG* was added to the intergenic region between convergently transcribed genes BF638R_0867 and 0868. For this, *ermG* with its upstream promoter was amplified from cloning vector pNBU2-*bla*-*ermG*. Regions flanking the BF638R_0867 and 0868 insertion site were cloned on each side of *ermG* in vector pLGB36 that was PCR amplified to remove its *ermG* gene. The correct plasmid was confirmed by whole plasmid sequencing and conjugally transferred into 638RΔT6SS with cointegrates and double cross-outs selected as described above.

Donor strain PvCL04Δ*ermG*pBCPT-*tetQ-ttr* and recipient strains 638RΔT6SS*ermG* and *B. ovatus* D2 were grown in basal medium until an OD_600_ of 0.7. 100 µl of donor and 20 µl of recipient (5:1 donor recipient) were mixed and 10 µl was plated on a BHIS plate for 20 h. The co-culture spot was cut from the plate and resuspended in 1 ml basal and 10-fold serial dilutions were plated on BHIS with erythromycin (number of recipients) or erythromycin and tetracycline (transconjugants). Transconjugants were passaged twice, and PCR was performed to confirm that the pBCPT transferred to the recipient strain. The multiplex PCR is based on the 16 S rRNA gene and produces different sized products for different Bacteroidales species^38^.

### His-DpnA., His-DpnB, His-BSAP-3, His-BcpT and site mutants

BcpT-encoding gene (CS034_04058) was PCR amplified removing the N-terminal signal sequence and ligated into expression vector pET16b (Novagen) to produce an N-terminal His-tag. Point mutations in this His-BcpT construct were generated via Quickchange mutagenesis (Stratagene) and confirmed by DNA sequencing. BSAP-3-encoding gene (HMPREF1058_01765) was PCR amplified removing the N-terminal signal sequence and cloned into NdeI digested pET16b. Dpn genes lacking signal peptidase II sequences with a C-terminal polyhistidine (6x) tag were codon optimized for expression in *E. coli* and cloned into pET21b+ via the NdeI and XhoI sites (Genscript).

Purification of polyhistidine-tagged proteins was performed as previously described^39^. Briefly, an overnight culture of *E. coli* BL21 (DE3) cells containing the plasmid encoding the protein of interest was used to inoculate 1.7 L culture of Terrific Broth (TB, EMD Millipore) supplemented with the appropriate antibiotics. The culture was induced via 0.5 mM isopropyl β-D-1-thiogalactopyranoside (IPTG, Gold Biosciences) at an OD_600_ ~1.0 and incubated at 37 °C for 5 h with the exception of Dnp2, which required expression at 18 °C, possibly due to it being proteolytically active on *E. coli* cells upon expression. The cells were spun down and resuspended in buffer A (10 mM MES pH 6.5, 150 mM NaCl) and the cells lysed by 3 passages through and Emulsiflex C3 cell disruptor (Avestin) at 15,000 psi. Cell debris was removed via centrifugation (14,000xg for 20 min) and the clarified lysate was recirculated in a column containing 10 ml of metal-chelating sepharose (GE) charged with cobalt. The polyhistidine-tagged proteins were eluted from the column using a 0-50% buffer B gradient (10 mM MES pH 6.5, 1 M imidazole, 90 mL total gradient volume). The fractions containing the purified protein were pooled, concentrated, exchanged into Tris buffered saline (TBS), pH 8.0 with 10% glycerol, and flash frozen in liquid nitrogen prior to storage at −80 °C.

### Enzymatic activation of BcpT and Proteases

Recombinant, purified BcpT was activated using Factor Xa (Millipore) following the supplier instructions. Briefly, a 1:200 Factor Xa to BcpT (w:w) ratio was added into TBS pH 8.0, 5 mM Ca^2++^ and incubated for 72 h at RT to achieve complete cleavage of BcpT, as measured by the absence of uncleaved BcpT detected via Western blot. Factor Xa was then removed from sample via supplied affinity beads. All FXa activated BcpT used as active toxin in this study was cleaved to completion using this method.

DpnA eluted as a proenzyme and presumably requires cleavage of a putative activation loop, similar to that found in fragipain^20^ for activity. Trypsin was added to DpnA at a 1:50 (w:w) ratio and incubated at 37 °C for 15 min. Trypsin was inactivated by bringing the sample to 1 mM phenylmethylsulphonyl fluoride (PMSF). Complete cleavage and proteolytic activation of BcpT was achieved by adding activated DpnA to BcpT at a 1:25 (w:w) ratio and incubating at room temperature for 2 h. DpnB eluted in an active form and did not require treatment with trypsin for activation. Proteolytic activation of BcpT by DpnB was achieved by adding activated DpnB to BcpT at a 1:25 ratio (w:w) and incubated at room temperature for 2 h.

His-BSAP-3 was activated using a 5:1 BSAP-3:DpnB ratio and incubating at 37 °C for 3 h in TBS, pH 8.0.

### BcpT site mutants for expression in PdCL02 Δ*bcpT*

To study the effect of mutation of R65 and R199 of BcpT when expressed from *P. dorei*, the R65N, R199N, and double R65N-R199N site mutants of *bcpT* were constructed using the His-tagged constructs as template (see above) removing the His-tag and adding the native ribosome binding site and signal sequence and expressed using pFD340. The native *bcpT* cloned into pFD340 was used as template for the rbs and the signal sequence, and the His-tagged clones were used as template for the native and three site mutants of BcpT. These pieces were cloned directionally into BamHI-digested pFD340 using NEBuilder. All plasmids were verified by whole plasmid sequencing and conjugally transferred to PdCL02 Δ*bcpT*.

### Antiserum generation and western blot analysis

Antiserum to BcpT was generated in rabbits at Lampire Biological Laboratories using the Expressline protocol with purified His-tagged BcpT as the immunogen. Use of rabbits for antiserum generation was approved by the Institutional Animal Care and Use Committee (IACUC), Brigham & Women’s Hospital and complies with all relevant ethical regulations for animal testing and research. Antibodies specific to His-BcpT were purified by conjugating 10 mg of purified recombinant His-BcpT to 1 ml of Affi-Gel 15 beads (Bio-Rad) following the manufacturer’s instructions. Rabbit anti-BcpT was recirculated through this affinity column for 30 min at room temperature. The beads were washed with 10 column volumes of 10 mM Tris, pH 8.0, 1 M NaCl and eluted using 50 mM glycine, pH 2.5. One ml fractions were collected into tubes containing 200 μL of 1 M Tris, pH 8.0 to neutralize the fractions. The antibody was dialyzed into HBS (50 mM HEPES, 100 mM NaCl, pH 7.4) and stored at 4 °C. The antiserum to the O-antigen of PvCL10 was generated previously^3^.

Proteins from SDS-PAGE gels were electrophoretically transferred to nitrocellulose or PVDF  and then blocked with blocking buffer (3% skim milk in blot buffer (100 mM Tris-HCl, pH8.0, 150 mM NaCl) for 1 h. Affinity-purified α-BcpT was then added at a 1:1000 dilution and incubated overnight at 4 °C with rocking or for 1 hr at room temperature. The membrane was then washed three times for 10 min with 30 ml of blot buffer + 0.1% Tween 20. Secondary antibody (α-rabbit IgG-HRP or alkaline phosphatase conjugate) was then added at a 1:500 dilution to 20 ml blot buffer + 0.1% Tween 20 and incubated for 1 h and then washed as above. Bands were identified by colorimetric assay or by chemiluminescence using ECL Western blotting reagents (Millipore) and imaged on Blublot HS film Life Science Products).

### N-terminal sequencing of BcpT fragments

His-tagged BcpT was treated with Factor Xa and the fragments were separated on 12% SDS-PAGE gel under non-reducing conditions. After transfer of the contents of the gel to a PVDF membrane, the membrane was washed in distilled water, strained with 0.02% Coomassie Brilliant blue in 40% methanol, 5% acetic acid for 20–30 s, destained in 40% methanol, 5% acetic acid for 1 min, and finally rinsed in distilled water. Fragments of ~35 kDa, 25 kDa and 15 kDa were cut from the membrane and sent for N-terminal sequencing at Tufts Core Facility. Five N-terminal residues were identified for each fragment.

### Fluorescent labeling of BcpT

Cysteine modification of BcpT was performed as previously described^40^. Briefly, BcpT was incubated with a 20-fold molar excess of Alexa Fluor 488 C_5_ maleimide (Invitrogen) overnight at 4 °C. Free dye was removed from the labeled sample using a G-50 Sephadex column (GE), and sample concentration was assessed via colorimetric assay. Absorbance of fluorophore at 495 nm was obtained and labeling efficiency was calculated using the molar extinction coefficient (73,000 cm^−1^M^−1^).

### Membrane purification

Cells for membrane purifications from various Bacteroidales strains were grown to OD_600_ = 0.8 and centrifuged at 14,000 × *g* for 30 min. Cells were resuspended in HBS (10 mM HEPES, pH 7.4, 150 mM NaCl) and passed through a cell disruptor (Avestin) 3 times at 15,000 psi. Cell debris was removed via centrifugation at 7000 × *g* for 10 min, and the supernatant was further centrifuged at 530,000 × *g *to pellet the membranes. Membranes were resuspended in HBS using a Dounce homogenizer.

### Purified LPS and membrane receptor blots

LPS was purified by the hot phenol method previously described^41^. Purified LPS from *E. coli* 0111:B4 and *Salmonella typhimurium* were purchased from List Labs. Purified membranes or LPS samples were separated on a 4-20% SDS-PAGE gradient gel prior to transfer to nitrocellulose membranes. Proteins and LPS were electrophoretically transferred to nitrocellulose, which were incubated with western blot blocking buffer prior to probing them with purified activated or unactivated BcpT (30 μg total at 1.5 μg/ml in 3% skim milk in blot buffer) with or without Alexa Fluor 488 label and incubated overnight at 4 °C. Blots incubated with fluorescently labeled protein were washed 3 times for 10 min in blot buffer containing 0.1% Tween 20 and imaged immediately using a Chemidoc 10 imaging system (BioRad). Blots treated with unlabeled protein were washed as above and then incubated with affinity-purified α-BcpT for 2 h at room temperature in blocking buffer. The blot was washed as above and then incubated with HRP conjugated secondary antibody (Invitrogen) in blot wash buffer for 1 h at room temperature. After washing the blot as described above the blots were visualized with ECL Western blotting reagents (Millipore) per the supplier’s instructions and imaged on film as described above for Western blots.

### Genome sequencing

The genomes of PvCL04 and PvCL05 were sequenced by the Biopolymers Facility, Harvard Medical School, Boston, MA, using the Illumina MiSeq platform, generating paired-end reads of 150 bp. Adapter sequences were removed, and the reads were quality trimmed using BBDuk (a component of the BBTools program suite distributed by the Department of Energy’s Joint Genome Institute; https://jgi.doe.gov/data-and-tools/bbtools/). Reads were screened for vector contamination using NCBI’s UniVecCore collection (build 10.0, with entries originating in GenBank removed) and reads returning a significant hit were discarded. De novo assembly was performed using Velvet Optimizer and Velvet. Gene calling was performed using Prodigal 2.6.3 and annotation was performed using a customized version of Prokka 1.14.6.

Long-read sequencing of PvCL10 was performed by SNPsaurus (Institute of Molecular Biology, 1318 Franklin Blvd, Room 273 Onyx Bridge, Eugene, OR) using the PacBio Seqel II HiFi platform, and short-read sequencing was performed by the Biopolymers Facility, Harvard Medical School, Boston, MA, using the Illumina MiSeq platform. Assembly of the genome was conducted using the Flye assembler (version 2.9^42^) The genome was further polished by mapping the Illumina reads and the PacBio HiFi (css) reads to the assembled genome using Snippy (v4.60, Torsten Seemann, University of Melbourne, Australia, https://github.com/tseemann/snippy) in successive rounds and correcting the variations found.

### Analysis of genomic and metagenomic datasets

Our locally curated set of 1434 Bacteroidales genomes (described in^43^) was modified by removing entries not identified to the species level to create a subset database comprising 1148 genomes. This database was utilized for detection (using Blastn 2.10.0 + ) of homologs to various segments of the 9,177 bp pBCPT plasmid: a backbone query comprising 5,058 bp (join(1..2280, 6340..9117)), and the 1,500 bp *bcpT* toxin gene sequence alone (CS034_04058, 4672..6171). Blastn returns for all queries were retained if the percent ID was ≥ 75% over on an alignment ≥ 500 bp (for the three gene query) or ≥1000 bp (for the backbone query).

Analyses of metagenomic datasets for the presence and geographical distribution of the *bcpT* gene utilized a collection of 15 publicly available human gut metagenomic datasets comprising samples collected from 1767 individuals. This metagenomic collection and the methodology used were previously described^43^.

### RNASeq analysis

For RNASeq analysis, triplicate cultures of PvCL10 were grown to an OD_600_ of 0.43 – 0.47 and BcpT was added at 7.5 µg/ml. Bacteria were harvested after 3 h of BcpT treatment when their OD_600_ was 0.080 – 0.082, compared to the untreated control which was OD_600_ 1.35. Triplicate cultures of untreated bacteria were grown and harvested at OD_600_ 0.80 – 0.84 as the untreated control samples. RNASeq of these six samples was performed by Novogene (South Plainfield, NJ) as 150 bp paired-end reads. For RNASeq analysis of PvCL10 tn79 and the WT PvCL10, bacteria were grown to an OD_600_ of 0.6 and harvested. RNA sequencing was performed by the Molecular Biology Core Facilities, Dana Farber Cancer Institute as 75-bp paired-end reads, with samples and controls provided as biological duplicates.

Reads from all samples were adapter- and quality trimmed using utilities included in the BBMap package of bioinformatics tools (v. 38.90) and mapped to PvCL10 using the Bowtie 2 short-read aligner (v. 2.4.2)^44^. SAMtools (v. 1.11)^45^ was used to convert the Bowtie 2 output to sorted and indexed BAM files, and these were compared to General Feature Format (GFF) files of the intervals of protein-coding domains from the appropriate genome using BEDtools (v. 2.30.0)^46^. Domains annotated as pseudogenes or as partial, truncated, or frameshifted genes were excluded. The read mapping results were evaluated for differential gene expression using both DESeq2 (v. 1.30.0)^47^ and edgeR (v. 3.32.1)^48^. We considered a gene differentially expressed if the absolute value of its fold change (FC) in expression level under experimental conditions differed from the control conditions by ≥2 and if the adjusted *p*-value (padj for DESeq2 and FDR for edgeR) was ≤ 0.05, as calculated by both statistical packages. In cases where DESeq2 returned NA due to read count outlier detection, edgeR calculations were relied on exclusively for determination of differential expression.

Cross comparison of the PvCL10 RNASeq data to the published Pv8482 RNASeq data of bacteroidetocin A treated cells^8^ proceeded by first finding the reciprocal best hits (RBH) between the proteomes of the two *P. vulgatus* isolates. RBH analysis utilized blastp (v. 2.11.0) using settings as suggested by^49^ (e.g. -e-value 10^−6^, -seg yes, -soft_masking true, and -comp_based_stats 0).

### Reporting summary

Further information on research design is available in the Nature Research Reporting Summary linked to this article.

## Supplementary information

Supplementary Information

Reporting Summary

Description of Additional Supplementary Files

Dataset 1

Dataset 2

Dataset 3

## Data Availability

*P. vulgatus* CL04T12C01 and *P. vulgatus* CL05T12C02 genome sequences were deposited in GenBank under BioProject accession number PRJNA415639. The *P. vulgatus* CL10T00C06 genome was deposited in GenBank under BioProject accession number PRJNA830856. RNASeq data for both the BcpT exposure experiments and the transposon mutant experiments were deposited as BAM files in the SRA, also linked to BioProject PRJNA830856. DGE values with statistics from the RNASeq analyses for all PvCL10 genes from the untreated versus BcpT-treated samples are provided in Supplementary Data 1. Source data are provided with this paper.

## Source data

Source Data
