# Supplementary material for: A proteolytically activated antimicrobial toxin encoded on a mobile plasmid of Bacteroidales induces a protective response
Source: Nat Commun. 2022 Jul 23;13:4258. doi: 10.1038/s41467-022-31925-w (PMC9308784; doi:10.1038/s41467-022-31925-w)
Supplement: Supplementary file 1 — Supplementary Information [file 41467_2022_31925_MOESM1_ESM.pdf]

**Supplementary Table 1. Comparison of proteins encoded by pBUN24 and pBCPT**

| pBUN24 | | | | | | | % similarity | pBCPT (PvCL04 gene designations) | | | | | | |
|---|---|---|---|---|---|---|---|---|---|---|---|---|---|---|
| **gene name** | **product** | **motifs** | **start** | **stop** | **bp** | **aa** | | **gene name** | **predicted function** | **motifs** | **start** | **stop** | **bp** | **aa** |
| repA | repA | PF01051 | 476 | 1,453 | 978 | 325 | 92.62% | CS034_04053 | repA | PF01051 | 475 | 1,452 | 978 | 325 |
| CDS_9 (orf2) | hypothetical protein | | 1,467 | 1,649 | 183 | 60 | 96.36% | CS034_04054 | hypothetical protein | | 1,466 | 1,657 | 192 | 63 |
| CDS_8 (orf1) | hypothetical protein | | 1,763 | 2,284 | 522 | 173 | 94.80% | CS034_04055 | hypothetical protein | | 1,759 | 2,280 | 522 | 173 |
| CDS_7 (orf7) | hypothetical protein | | 2,314 | 2,855 | 542 | 180 | | CS034_04056 | LuxR-type DNA-binding HTH domain protein | PF00196 | 2,301 | 3,938 | 1,638 | 545 |
| CDS_6 (orf6) | HTH transcriptional regulator | | 2,878 | 3,975 | 1,098 | 365 | | CS034_04057 | predicted OMP | | 4,086 | 4,658 | 573 | 190 |
| CDS_5 (orf5) | putative lipoprotein | PF08450 | 4,195 | 5,850 | 1,656 | 551 | | CS034_04058 | BcpT (experimental evidence) | | 4,672 | 6,171 | 1,500 | 499 |
| | | | | | | | | CS034_04059 | BcpI (experimental evidence) | | 6,228 | 6,401 | 174 | 57 |
| mobB | MobB | | 6,405 | 7,283 | 879 | 292 | 98.29% | CS034_04060 | MobB | DUF5712, PF18976 | 6,579 | 7,457 | 879 | 292 |
| mobA | MobA | | 7,280 | 7,885 | 606 | 201 | 99.50% | CS034_04061 | MobA | PF01726 | 7,454 | 8,059 | 606 | 201 |
| CDS_4 (orf4) | hypothetical protein | | 7,917 | 8,183 | 267 | 88 | | | | | | | | |
| CDS_3 (tad) | TA system toxin | PF05973, TIGR03070 | 8,277 | 8,624 | 348 | 115 | 98.26% | CS034_04062 | HipB-like toxin | PF05973, TIGR03070 | 8,450 | 8,797 | 348 | 115 |
| CDS_2 (ata) | TA system anti-toxin | PF15731 | 8,630 | 8,944 | 315 | 104 | 100.00% | CS034_04063 | MqsA-like anti-toxin | PF15731 | 8,803 | 9,117 | 315 | 104 |
| CDS_1 (orf3) | hypothetical protein | | 8,938 | 263 | 270 | 89 | | | | | | | | |

**a.** CDS names are from NCBI GenBank EU818711 file, pBUN24 names are from Fig 1 in **Shkoporov AN, et al.** 2013. Analysis of a novel 8.9kb cryptic plasmid from *Bacteroides uniformis* , its long-term stability and spread within human microbiota. Plasmid. **69(2):**146-59.

Yellow highlighted genes are those unique between each of the two plasmids

**Supplementary Table 2**. Primers used in this study

| | | |
|---|---|---|
| Construction of PvCL04 and PdCL02 Δ*bcpT* | left flank forward | caagggatccataagaaaaaggcacatcgagaac |
| | left flank reverse | gtcgacgcgtttgttacatgaacacaaaatagctg |
| | right flank forward | atgtacgcgttttgttggtaagccagaaattgta |
| | right flank reverse | cgaaggatccaagatcttctcctatatcgcaagc |
| Clone *bcpT* into pFD340 | forward | accaggatccaaacacggtataacaaaagaacca |
| | reverse | aggaggatccactccttccctattttgtttagga |
| Screening for *bcpT* in unsequenced strains | forward | ggtatatactatggagttgc |
| | reverse | actccttccctattttgtttagga |
| Outward primers for circular plasmid confirmation | left junction primer | gtggatatttgttttccaacatga |
| | right junction primer | aagataaagcgtatggaagcaaag |
| Construction of His-BcpT for expression in *E. coli* | forward | acatcatatgaacaatgaacttcctattgaaaatgtaca |
| | reverse | aggaggatccactccttccctattttgtttagga |
| His-BcpT (R65N) | forward | gataatcctattttaacaaacgcaagttcatcttttg |
| | reverse | caaaagatgaacttgcgtttgttaaaataggattatc |
| His-BcpT (R199N) | forward | gggactgaaactctcacaaactccgcaagcgatggaggt |
| | reverse | acctccatcgcttgcggagtttgtgagagtttcagtccc |
| Amplify native and site mutants of *bcpT* from His-constructs | forward | gtgttcatgtaacaatgaacttcctattgaaaat |
| | reverse | attcgagctcggtacccgggactccttccctattttgtttag |
| Amplify rbs and signal sequence of *bcpT* for construction of *bcpT* site mutants in pFD340 | forward | aatcagaattgactctagagcacggtataacaaaagaacc |
| | reverse | gttcattgttacatgaacacaaaatagctg |
| Construction of PdCL02 Δ*dpn1* (HMPREF1064_01439) BamHI digested pLGB13 | left flank forward | taagattagcattatgagtggcatctaagatagctgac |
| | left flank reverse | ctttataccagccacatactaataacagaaaac |
| | right flank forward | agtatgtggctggtataaagatactaatacacattagag |
| | right flank reverse | cgaattcctgcagcccgggggctaactcagatgctcgc |
| Construction of PdCL02 Δ*dpn2* (HMPREF1064_00451) BamHI digested pLGB13 | left flank forward | taagattagcattatgagtgggtaggctcgttgaacaag |
| | left flank reverse | cagcccatcccataagaaaaggatcactcttcc |
| | right flank forward | ttttcttatgggatgggctgaaacggaatc |
| | right flank reverse | cgaattcctgcagcccggggaagcaatgatggctctttctatc |
| Complement PdCL02 Δ*dpn2* (in pFD340) | forward | aatcagaattgactctagagtcaagaaaccggaagagtg |
| | reverse | attcgagctcggtacccgggctggctgcattctccttc |
| Deletion of PvCL10 M0N98_03761-60 (sigma factor/antisigma factor) | left flank forward | cggtgtaagattagcattatgagtgtccgcttcatggagactc |
| | left flank reverse | agcaatgacacggtgagcttgcacgtacaaac |
| | right flank forward | acgtgcaagctcaccgtgtcattgctccttc |
| | right flank reverse | gatatcgaattcctgcagcccgggggtttcgcggtcactcatc |
| Clone M0N98_03761 into pFD340 | forward | ttctggatcccggatgcagtattttttgcagtctt |
| | reverse | ctacggatccggttccgaatagccctccatc |
| Clone M0N98_03760 into pFD340 | forward | gatgggatcctacagagccaaaaccttattgatg |
| | reverse | tcatggatccattttatataagaacagtgaaaaagttcat |
| Clone M0N98_03761-60 into pFD340 | forward | ttctggatcccggatgcagtattttttgcagtctt |
| | reverse | tcatggatccattttatataagaacagtgaaaaagttcat |
| Construction of Pv8482 O-ag deletion mutant Bvu_1068-69 cloned into PstI site pLGB13 | left flank forward | agtggatcccccgggctgcaccaaaatagggcacgttatatc |
| | left flank reverse | tctttatgattacctccagtaccggatattc |
| | right flank forward | actggaggtaatcataaagaaatggatggaagtgatc |
| | right flank reverse | cttgatatcgaattcctgcagtggatgatccggagctg |
| Construction of His-BSAP-3 for expression in *E. coli* | forward | taaacatatgtgtacaaatacttatgacgtttgtgaaat |
| | reverse | ccaccatatgttatggataaacatatcctaaaataccacc |
| Construction of PvCL04 *ermG* deletion mutant into BamHI site pLGB30 | left flank forward | taagattagcattatgagtgcgtaggcaaaaacggcatag |
| | left flank reverse | ctttgaactacattagtaacttcttacaggtgaatac |
| | right flank forward | gttactaatgtagttcaaagtcgggtgg |
| | right flank reverse | cgaattcctgcagcccggggcgacacggttatttgggatag |

| | | |
|---|---|---|
| **Construction of pBCPT-*tetQ*** | | |
| PCR amplify BcCL03T12C61 *tetQ* | forward | ttcgtcgattcttgccagttgaacctac |
| | reverse | aattctgttcccgtattgccttatagaaatttc |
| PCR amplify BF638R_1994 transcriptional stop | forward | ggcaatacgggaacagaattggccgaag |
| | reverse | actgccgagcccaccaattccatattcaag |
| PCR amplify pBCPT flanks for *tetQ* integration | left flank forward | taagattagcattatgagtgggtcgaacttataacgggc |
| | left flank reverse | aactggcaagaatcgacgaacaccctcc |
| | right flank forward | gaattggtgggctcggcagtcgaaagaac |
| | right flank reverse | cgaattcctgcagcccgggggtgacctcccttgcgctc |
| **Insertion of *ermG* in *B. fragilis* 638RΔT6SS** | | |
| PCR amplify *ermG* from pNBU2-*ermG* | forward | tcttctaaccgcttgacggaaatcaacaaattttag |
| | reverse | ttgcactacgcagtttgatttctcaggactttac |
| PCR amplify flanking regions to insert *ermG* | left flank forward | gctcaacaattgcttgacgggtggagctgatgcgtttc |
| | left flank reverse | tccgtcaagcggttagaagaattgtggaagtc |
| | right flank forward | aatcaaactgcgtagtgcaaatatagcg |
| | right flank reverse | tgcctgttctatttccgaaccgatgtagccattatcgatg |
| amplify pLGB36 removing plasmid *ermG* | forward | gttcggaaatagaacagg |
| | reverse | ccgtcaagcaattgttgag |
| Detect pBCPT in transconjugants | forward | aatcagaattgactctagagcgctccttcccttcacttattg |
| | reverse | attcgagctcggtacccgggcattggtgatgatgctatttaagatag |
| forward multiplex primer (for all three species) | MPI forward | gtacacaccgcccgt |
| multiplex *P. vulgatus* reverse | | ctttctctcttccgtatcattac |
| Multiplex *B. fragilis* reverse | MPI-Bf reverse | gctaatcccccaatcatac |
| multiplex *B. ovatus* reverse | MPI-Bo reverse | atcaatattgcgtactcgaac |
| Clone PvCL04 CS034_04056 into pFD340 | forward | aatcagaattgactctagagctgagttgtaatcaaacctattg |
| | reverse | attcgagctcggtacccggggatagttaaggtgcatcac |
| Clone PvCL04 CS034_04057 into pFD340 | forward | aatcagaattgactctagaggtctaactttgcaacgttc |
| | reverse | attcgagctcggtacccgggttaaaatgaatatccaaaactaatataagg |
| Clone PvCL04 CS034_04059 into pFD340 | forward | aatcagaattgactctagagcgctccttcccttcacttattg |
| | reverse | attcgagctcggtacccgggcattggtgatgatgctatttaagatag |

**Restriction sites are underlined**

| Supplementary Table 3.  Strains used in this study | source | toxin phenotype of wild type Pv and Pd strains |
|---|---|---|
| *Phocaeicola dorei* CL02T00C15 | Comstock lab (Zitomersky, 2011) | BcpT |
| *Phocaeicola dorei* CL02T00C15 Δ*bcpT* (ΔHMPREF1063_05166 ) | this study | |
| *Phocaeicola dorei* CL02T00C15 Δ*bcpT* p*bcpT* | this study | |
| *Phocaeicola dorei* CL02T00C15 Δ*bcpT* p*bcpT* R65N | this study | |
| *Phocaeicola dorei* CL02T00C15 Δ*bcpT* p*bcpT* R199N | this study | |
| *Phocaeicola dorei* CL02T00C15 Δ*bcpT* p*bcpT* R65N R199N | this study | |
| *Phocaeicola vulgatus* CL04T12C01 | Comstock lab (Zitomersky, 2011) | BcpT |
| *Phocaeicola vulgatus* CL04T12C01 tn10 | this study | |
| *Phocaeicola vulgatus* CL04T12C01 tn12 | this study | |
| *Phocaeicola vulgatus* CL04T12C01 Δ*bcpT* (ΔCS034_04058) | this study | |
| *Phocaeicola vulgatus* CL04T12C01 Δ*bcpT* p*bcpT* | this study | |
| *Phocaeicola vulgatus* CL04T12C01 *ΔermG* pBCPT-*tetQ-ttr* | this study | |
| *Phocaeicola dorei* CL03T12C01 | Comstock lab (Zitomersky, 2011) | BSAP-3 |
| *Phocaeicola dorei* 9_1_42 FAA | BEI | BSAP-3 |
| *Phocaeicola dorei* 5_1_36/D4 | BEI | |
| *Phocaeicola dorei* CL01T00C33 | Comstock lab (Zitomersky, 2011) | |
| *Phocaeicola dorei* CL08T03C21 | Comstock lab (Zitomersky, 2011) | |
| *Phocaeicola dorei* CL12T00C02 | Comstock lab (Zitomersky, 2011) | |
| *Phocaeicola dorei* CL15T00C36 | Comstock lab (Zitomersky, 2011) | |
| *Phocaeicola vulgatus* ATCC 8482 | ATCC | |
| *Phocaeicola vulgatus* ATCC 8482 Δ*Bvu_1068-69* (O-ag mutant) | this study | |
| *Phocaeicola vulgatus* CL05T12C02 | Comstock lab (Zitomersky, 2011) | BcpT |
| *Phocaeicola vulgatus* CL06T12C01 | Comstock lab (Zitomersky, 2011) | |
| *Phocaeicola vulgatus* CL07T06C01 | Comstock lab (Zitomersky, 2011) | |
| *Phocaeicola vulgatus* CL09T03C04 | Comstock lab (Zitomersky, 2011) | BSAP-3 |
| *Phocaeicola vulgatus* CL10T00C06 | Comstock lab (Zitomersky, 2011) | |
| *Phocaeicola vulgatus* CL10T00C06 Δ*CK234_00400 - 401* (O-ag mutant) | Comstock lab (Laclare McEneany, 2018) | |
| *Phocaeicola vulgatus* CL10T00C06 Δ*CK234_00923-924* (Δsig/antisig) | this study | |
| *Phocaeicola vulgatus* CL10T00C06 Δ*CK234_00923-924* p*00923* | this study | |
| *Phocaeicola vulgatus* CL10T00C06 Δ*CK234_00923-924* p*00924* | this study | |
| *Phocaeicola vulgatus* CL10T00C06 pBCPT *gene (CS034_04056)* | this study | |
| *Phocaeicola vulgatus* CL10T00C06 pBCPT *gene (CS034_04057)* | this study | |
| *Phocaeicola vulgatus* CL10T00C06 pBCPT *gene (CS034_04059)* | this study | |
| *Phocaeicola vulgatus* CL11T12C09 | Comstock lab (Zitomersky, 2011) | |
| *Phocaeicola vulgatus* CL13T00C01 | Comstock lab (Zitomersky, 2011) | |
| *Phocaeicola vulgatus* CL14T03C19 | Comstock lab (Zitomersky, 2011) | bacteroidetocin A, BcpT |
| *Bacteroides thetaiotamicron* VPI 5482 | lab stock | |
| *Bacteroides thetaiotamicron* VPI 5482 pFD340 | Comstock lab (Laclare McEneany, 2018) | |
| *Bacteroides thetaiotamicron* VPI 5482 p*bcpT* | this study | |
| *Bacteroides ovatus* D2 | BEI | |
| *Bacteroides ovatus* D2 pBCPT-*tetQ-ttr* | this study | |
| *Phocaeicola dorei* CL02T12C06 | Comstock lab (Zitomersky, 2011) | BcpT |
| *Phocaeicola dorei* CL02T12C06 Δ*dpn1* | this study | |
| *Phocaeicola dorei* CL02T12C06 Δ*dpn2* | this study | |
| *Phocaeicola dorei* CL02T12C06 Δ*dpn1* Δ*dpn2* | this study | |
| *Bacteroides fragilis* 638R | lab stock | |
| *Bacteroides fragilis* 638RΔT6SS*ermG* | this study | |
| *Bacteroides fragilis* 638RΔT6SS*ermG* pBCPT-*tetQ-ttr* | this study | |
| *Bacteroides ovatus* ATCC 8483 | ATCC | |
| *Bacteroides uniformis* ATCC 8492 | ATCC | |
| *Bacteroides finegoldii* CL09T03C10 | Comstock lab (Zitomersky, 2011) | |
| *Parabacteroiees merdae* CL03T12C32 | Comstock lab (Zitomersky, 2011) | |
| *Parabacteroides johnsonii* CL02T12C29 | Comstock lab (Zitomersky, 2011) | |
| *Parabacteroides goldsteinii* CL02T12C30 | Comstock lab (Zitomersky, 2011) | |
| *E. coli* DH5α | lab collection | |
| *E. coli* BL21/DE3 | lab collection | |
| *E. coli* S17 λ pir | E. Martens | |
| *E. coli* HB101 | lab collection | |
| *E. coli* HS | lab collection | |
| *E. coli* BL21/DE3 pHis-*bcpT* | this study | |
| *E. coli* BL21/DE3 pHis-*bcpT* R65N | this study | |

| | | |
|---|---|---|
| *E. coli* BL21/DE3 pHis-*bcpT* R199N | this study | |
| *E. coli* BL21/DE3 pHis-*bcpT* R65N R199N | this study | |
| *E. coli* BL21/DE3 pHis-*dpn1* | this study | |
| *E. coli* BL21/DE3 pHis-*dpn2* | this study | |
| *Salmonella enterica* ATCC 14028 | ATCC | |


Zitomersky NL, Coyne MJ, Comstock LE. Longitudinal analysis of the prevalence, maintenance, and IgA response to species of the order Bacteroidales in the human gut. Infect Immun. 2011 May;79(5):2012-20. doi: 10.1128/IAI.01348-10. Epub 2011 Mar 14. PMID: 21402766; PMCID: PMC3088145.

McEneany VL, Coyne MJ, Chatzidaki-Livanis M, Comstock LE. Acquisition of MACPF domain-encoding genes is the main contributor to LPS glycan diversity in gut Bacteroides species. ISME J. 2018 Dec;12(12):2919-2928. doi: 10.1038/s41396-018-0244-4. Epub 2018 Jul 31. PMID: 30065309; PMCID: PMC6246601.

Roelofs KG, Coyne MJ, Gentyala RR, Chatzidaki-Livanis M, Comstock LE. Bacteroidales Secreted Antimicrobial Proteins Target Surface Molecules Necessary for Gut Colonization and Mediate Competition In Vivo. mBio. 2016 Aug 23;7(4):e01055-16. doi: 10.1128/mBio.01055-16. PMID: 27555309; PMCID: PMC4999547.

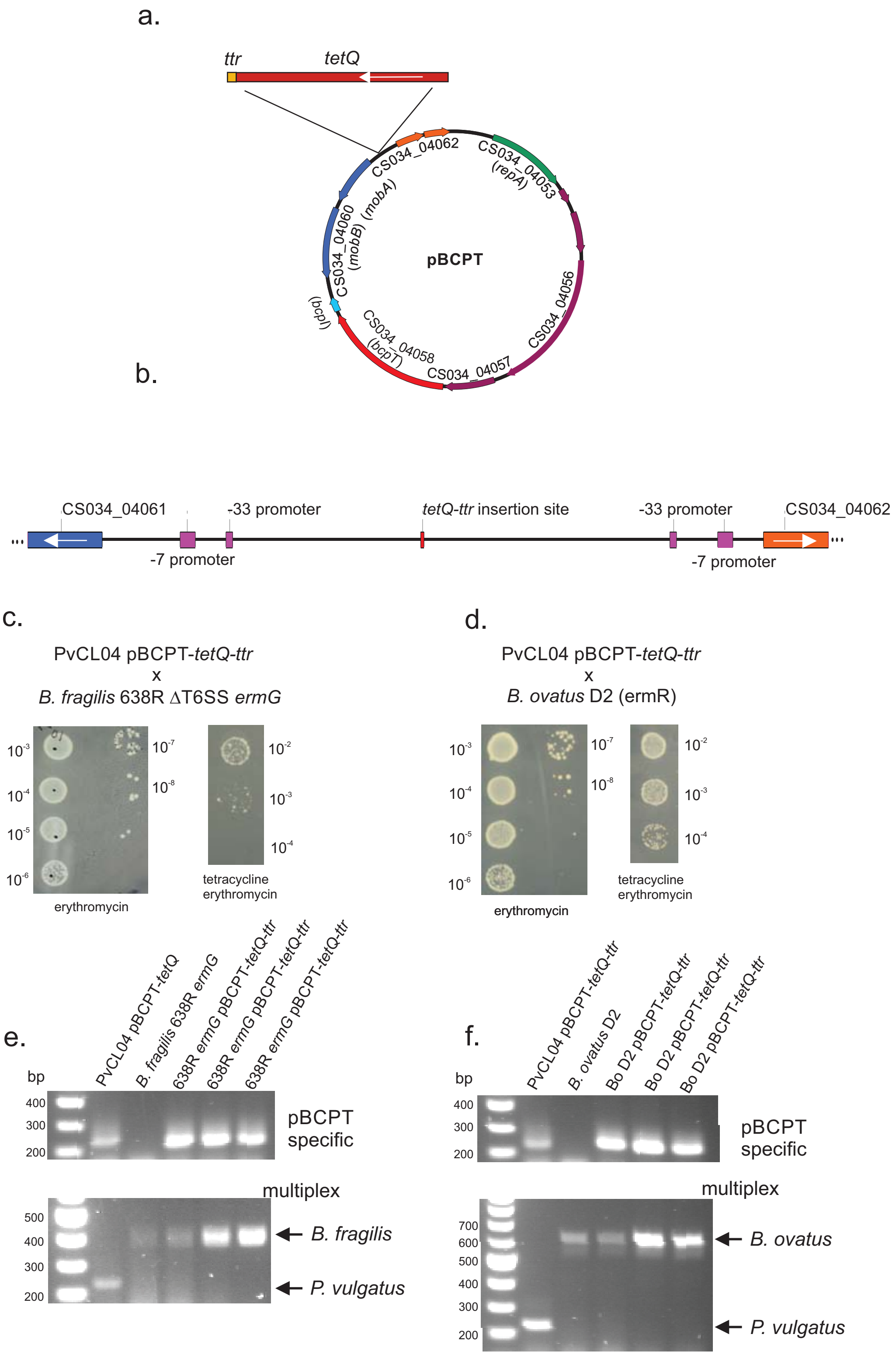


**Supplementary Figure 1. Analysis of pBCPT plasmid mobilization.** **a.** Plasmid map of pBCPT with gene names from PvCL04. The insertion site of *tetQ* and the transcriptional termination region (*ttr*) is shown. **b.** Amplified intergenic region between CS034_04061 and CS034_04061 showing that the insertion does not affect the promoters of either divergently transcribed gene. **c. d.** Plates showing the colonies arising on erythromycin plates (recipient strain) and tetracycline/erythromycin plates (transconjugants) for transfer of the pBCPT-*tetQ* plasmid from PvCL04 to *B. fragilis* 638R*ermG*ΔT6SS (C) and *B. ovatus* D2 (D). **e. f.** Amplicons resulting from PCR with primers specific to the *bcpT* gene (top) and a multiplex of the 16S rRNA gene (bottom) of the transconjugants.

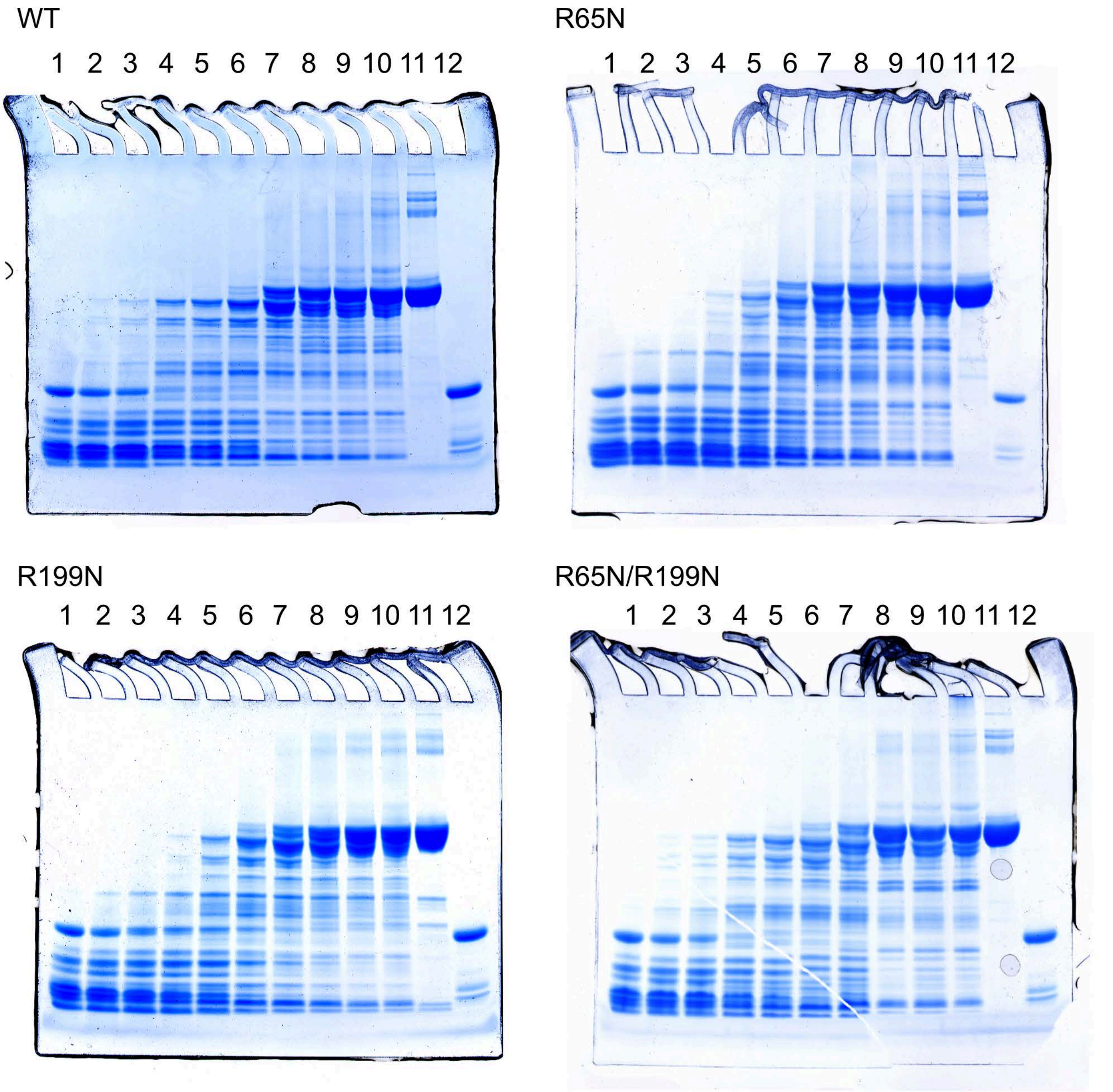


**Figure S2. Trypsin digestion of BcpT cleavage site mutants**. Purified recombinant wt BcpT and its cleavage site mutants, R65N, R199N and R65N-R199N, were cleaved (5µg in each reaction) with chymotrypsin. Two-fold dilutions of chymotrypsin starting with 2.5 µg of chymotrypsin were generated in HBS and to each dilution 5µg of BcpT was added (final reaction volume of 20µl) and incubated for 30 min at 37°C. The digestion reactions were stopped by the addition of 5µl of SDS-PAGE sample buffer and heating to 95°C for 5 min. The cleavage products were separated on an 8-16% SDS-PAGE: the cleavage products are shown in lanes 1-10. Lane 11 contains uncleaved WT BcpT or its derivatives. Lane 12 contains 2.5 µg of chymotrypsin.

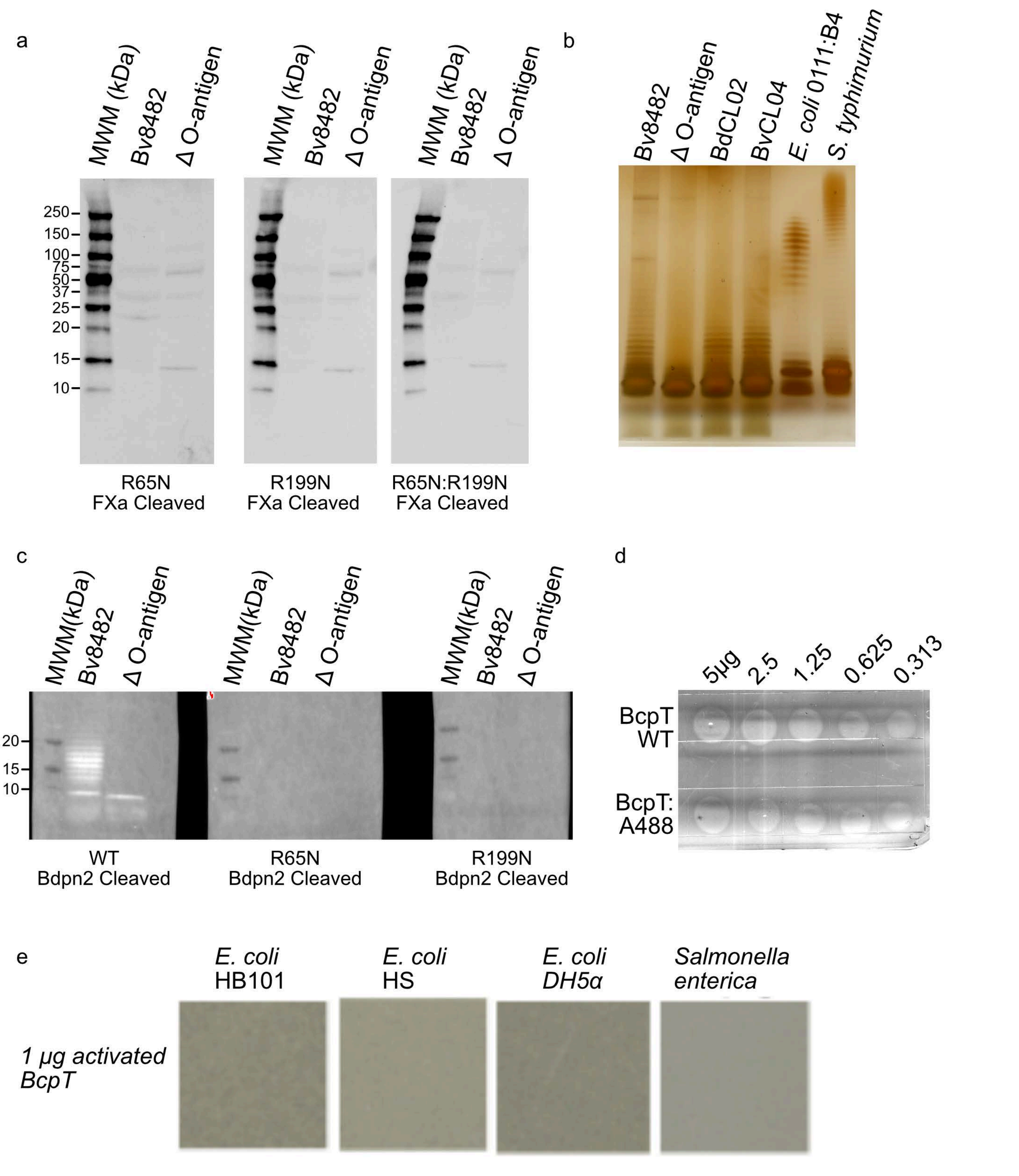


**Figure S3. Analysis of BcpT and BcpT site mutants binding to components of membrane fraction and LPS of indicated strains on Western blots.** **a**. Receptor blot of membranes from Bv8482 or its O-antigen mutant probed with BcpT site mutants (see Methods for receptor blots details). **b**. Silver-stain analysis of LPS extracts from indicated strains. **c.** Probing of blots containing purified LPS with Alexa-488-labeled BcpT. **d**. Spot tests of the activated WT BcpT and the Alexa-fluor labeled WT BcpT use to probe the lipid blots in panel e and in Fig. 5. **e**. Spot tests of activated BcpT (5µg) on the strains of *E. coli* and *Salmonella enterica*.

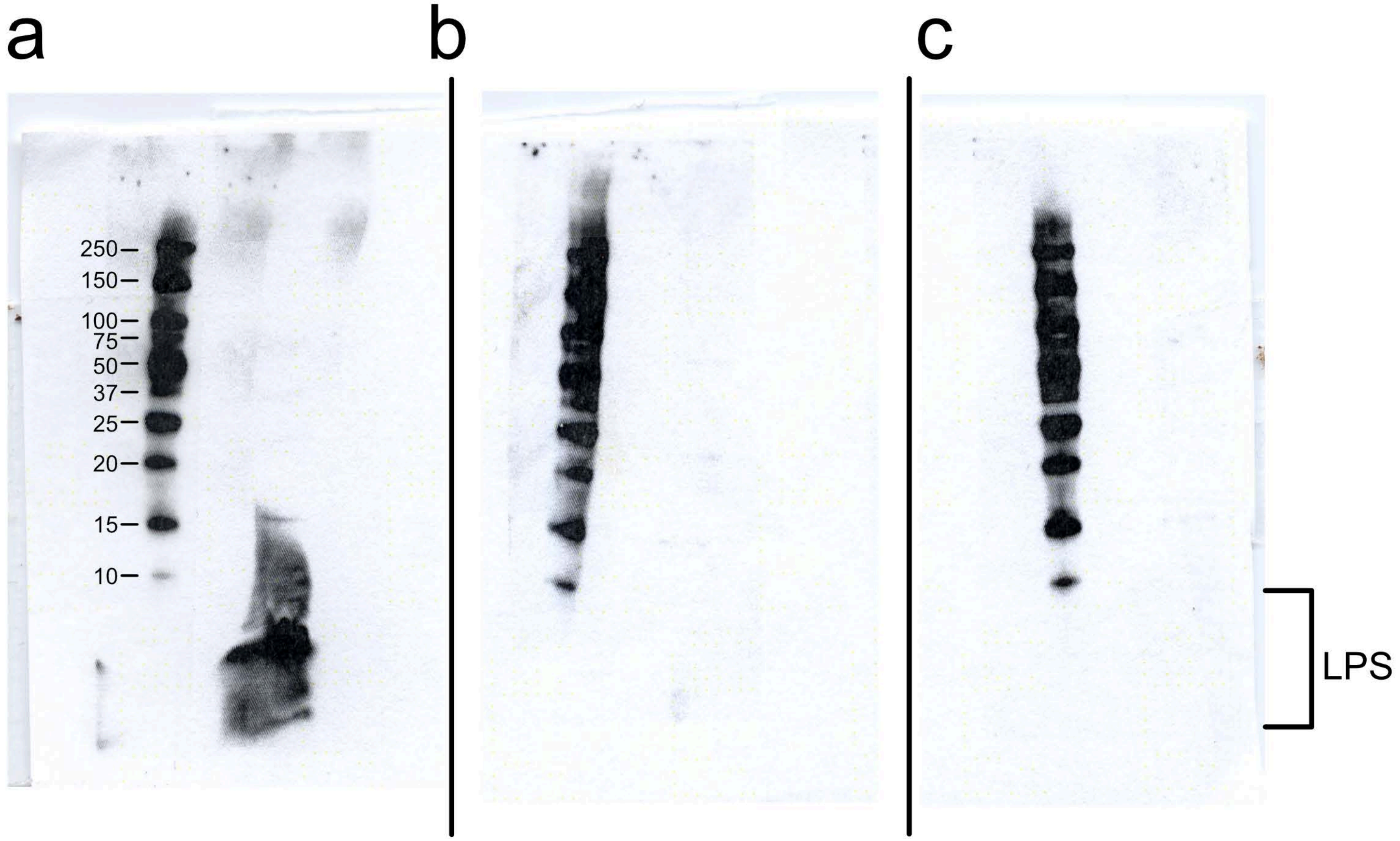


**Figure S4. Amino terminal fragments of BcpT block BcpT binding to membrane LPS. a**. Receptor blot of Bv8482 membranes probed with FXa-cleaved BcpT (1.5μg/ml) (see Methods for details). **b**. Receptor blot of Bv8482 membranes probed with FXa-cleaved BcpT in the presence of 20-fold molar excess N-terminal BcpT fragment 18-199. **c**. Receptor blot of Bv8482 membranes probed with FXa-cleaved BcpT in the presence of 20-fold molar excess N-terminal BcpT fragment 66-199.

Supplemental references

1) Shkoporov, A. N. *et al.* Analysis of a novel 8.9kb cryptic plasmid from *Bacteroides uniformis*, its long-term stability and spread within human microbiota. *Plasmid* **69**, 146-159, doi:10.1016/j.plasmid.2012.11.002 (2013).

2) Zitomersky NL, Coyne MJ, Comstock LE. Longitudinal analysis of the prevalence, maintenance, and IgA response to species of the order Bacteroidales in the human gut. Infect Immun. 2011 May;79(5):2012-20. doi: 10.1128/IAI.01348-10. Epub 2011 Mar 14. PMID: 21402766; PMCID: PMC3088145.

3) Laclare McEneany, V., Coyne, M. J., Chatzidaki-Livanis, M. & Comstock, L. E. Acquisition of MACPF domain-encoding genes is the main contributor to LPS glycan diversity in gut *Bacteroides* species. *ISME J* **12**, 2919-2928, doi:10.1038/s41396-018-0244-4 (2018).

4) Roelofs, K. G., Coyne, M. J., Gentyala, R. R., Chatzidaki-Livanis, M. & Comstock, L. E. Bacteroidales Secreted Antimicrobial Proteins Target Surface Molecules Necessary for Gut Colonization and Mediate Competition In Vivo. *mBio* **7**, doi:10.1128/mBio.01055-16 (2016).
