# Supplementary material for: A proteolytically activated antimicrobial toxin encoded on a mobile plasmid of Bacteroidales induces a protective response
Source: Nat Commun. 2022 Jul 23;13:4258. doi: 10.1038/s41467-022-31925-w (PMC9308784; doi:10.1038/s41467-022-31925-w)
Supplement: Supplementary file 2 — Reporting Summary [file 41467_2022_31925_MOESM2_ESM.pdf]

nature portfolio

Corresponding author(s): Laurie Comstock/ Rodney Tweten

Last updated by author(s): Jun 30, 2022

Reporting Summary

Nature Portfolio wishes to improve the reproducibility of the work that we publish. This form provides structure for consistency and transparency in reporting. For further information on Nature Portfolio policies, see our Editorial Policies and the Editorial Policy Checklist.

Statistics

For all statistical analyses, confirm that the following items are present in the figure legend, table legend, main text, or Methods section.

n/a	Confirmed	
☐	☒	The exact sample size (*n*) for each experimental group/condition, given as a discrete number and unit of measurement
☐	☒	A statement on whether measurements were taken from distinct samples or whether the same sample was measured repeatedly
☐	☒	The statistical test(s) used AND whether they are one- or two-sided<br>*Only common tests should be described solely by name; describe more complex techniques in the Methods section.*
☒	☐	A description of all covariates tested
☒	☐	A description of any assumptions or corrections, such as tests of normality and adjustment for multiple comparisons
☒	☐	A full description of the statistical parameters including central tendency (e.g. means) or other basic estimates (e.g. regression coefficient) AND variation (e.g. standard deviation) or associated estimates of uncertainty (e.g. confidence intervals)
☐	☒	For null hypothesis testing, the test statistic (e.g. *F*, *t*, *r*) with confidence intervals, effect sizes, degrees of freedom and *P* value noted<br>*Give P values as exact values whenever suitable.*
☒	☐	For Bayesian analysis, information on the choice of priors and Markov chain Monte Carlo settings
☒	☐	For hierarchical and complex designs, identification of the appropriate level for tests and full reporting of outcomes
☒	☐	Estimates of effect sizes (e.g. Cohen's *d*, Pearson's *r*), indicating how they were calculated

Our web collection on statistics for biologists contains articles on many of the points above.

Software and code

Policy information about availability of computer code

Data collection

For genome sequences, adapter sequences were removed and the reads were quality trimmed using BBDuk (a component of the BBTools program suite distributed by the Department of Energy's Joint Genome Institute; https://jgi.doe.gov/data-and-tools/bbtools/)(v. 38.90) . Reads were screened for vector contamination using NCBI's UniVecCore collection (build 10.0, with entries originating in GenBank removed), and reads returning a significant hit were discarded. De novo assembly was performed using Velvet Optimizer and Velvet. Gene calling was performed using Prodigal 2.6.3 and annotation was performed using a customized version of Prokka 1.14.6.

Long-read sequencing of PvCL10 was performed by SNPsaurus (Institute of Molecular Biology, 1318 Franklin Blvd, Room 273 Onyx Bridge, Eugene, OR) using the PacBio Seqel II HiFi platform, and short-read sequencing was performed by the Biopolymers Facility, Harvard Medical School, Boston, MA, using the Illumina MiSeq platform. Assembly of the genome was conducted using the Flye assembler (version 2.944) The genome was further polished by mapping the Illumina reads and the PacBio HiFi (css) reads to the assembled genome using Snippy (v4.60, Torsten Seemann, University of Melbourne, Australia, https://github.com/tseemann/snippy) in successive rounds and correcting the variations found.

For RNASeq analysis, reads from all samples were adapter- and quality-trimmed using utilities included in the BBMap package of bioinformatics tools (v. 38.90) and mapped to PvCL10 using the Bowtie 2 short read aligner (v. 2.4.2)46. SAMtools (v. 1.11)47 was used to convert the Bowtie 2 output to sorted and indexed BAM files, and these were compared to General Feature Format (GFF) files of the intervals of protein-coding domains from the appropriate genome using BEDtools (v. 2.30.0)48. Domains annotated as pseudogenes or as partial, truncated, or frameshifted genes were excluded. The read mapping results were evaluated for differential gene expression using both DESeq2 (v. 1.30.0)49 and edgeR (v. 3.32.1)50. We considered a gene differentially expressed if the absolute value of its fold change (FC) in expression level under experimental conditions differed from the control conditions by ≥ 2 and if the adjusted p-value (padj for DESeq2 and FDR for edgeR) was ≤ 0.05, as calculated by both statistical packages. In cases where DESeq2 returned NA due to read count outlier detection, edgeR calculations were relied on exclusively for determination of differential expression.

Analysis of genomic and metagenomic datasets
Our locally curated set of 1434 Bacteroidales genomes (described in45) was modified by removing entries not identified to the species level to

create subset database comprising 1148 genomes. This database was utilized for detection (using Blastn 2.10.0+) of homologs to various segments of the 9,177 bp pBCPT plasmid

Data analysis	For genome sequences - Adapter sequences were removed and the reads were quality trimmed using BBDuk (a component of the BBTools program suite distributed by the Department of Energy's Joint Genome Institute; https://jgi.doe.gov/data-and-tools/bbtools/)(v. 38.90) . Reads were screened for vector contamination using NCBI's UniVecCore collection (build 10.0, with entries originating in GenBank removed) reads returning a significant hit were discarded. De novo assembly was performed using Velvet Optimizer and Velvet. Gene calling was performed using Prodigal 2.6.3 and annotation was performed using a customized version of Prokka 1.14.6. Blastp and Blastn 2.10.0+ was used for homology searches. For RNASeq analyses, Reads from all samples were adapter- and quality-trimmed using utilities included in the BBMap package of bioinformatics tools (v. 38.90) and mapped to using the Bowtie 2 short read aligner (v. 2.4.2)36. SAMtools (v. 1.11)37 was used to convert the Bowtie 2 output to sorted and indexed BAM files, and these were compared to General Feature Format (GFF) files of the intervals of protein-coding domains from the appropriate genome using BEDtools (v. 2.30.0)38. The read mapping results were evaluated for differential gene expression using both DESeq2 (v. 1.30.0)39 and edgeR (v. 3.32.1). Reciprocal best hit anaysis (RBH) analysis utilized blastp (v. 2.11.0) using settings as suggested by41 (e.g. -evalue 10-6, -seg yes, -soft_masking true, and -comp_based_stats 0).

For manuscripts utilizing custom algorithms or software that are central to the research but not yet described in published literature, software must be made available to editors and reviewers. We strongly encourage code deposition in a community repository (e.g. GitHub). See the Nature Portfolio guidelines for submitting code & software for further information.

Data

Policy information about availability of data

All manuscripts must include a data availability statement. This statement should provide the following information, where applicable:

- Accession codes, unique identifiers, or web links for publicly available datasets
- A description of any restrictions on data availability
- For clinical datasets or third party data, please ensure that the statement adheres to our policy

P. vulgatus CL04T12C01 and P. vulgatus CL05T12C02 genome sequences were deposited in GenBank under BioProject accession number PRJNA415639. The P. vulgatus CL10T00C06 genome was deposited in GenBank under BioProject accession number PRJNA830856. RNASeq data for both the BcpT exposure experiments and the transposon mutant experiments were deposited as BAM files in the SRA, also linked to BioProject PRJNA830856. DGE values with statistics from the RNASeq analyses for all PvCL10 genes from the untreated versus BcpT treated samples are provided in Supplementary Data 1.

Field-specific reporting

Please select the one below that is the best fit for your research. If you are not sure, read the appropriate sections before making your selection.

[x] Life sciences [ ] Behavioural & social sciences [ ] Ecological, evolutionary & environmental sciences

For a reference copy of the document with all sections, see nature.com/documents/nr-reporting-summary-flat.pdf

Life sciences study design

All studies must disclose on these points even when the disclosure is negative.

Sample size	The only experiments where statistics are provided is the RNASeq data and these were biological triplicates which our previous analyses have shown to be adequate to discern differences in two isogenic strains or strains under different growth conditions
Data exclusions	none
Replication	All reported phenotypes were reproduced multiple times as described in the figure legend with no outlying data
Randomization	not applicable ss these are not clinical studies
Blinding	these are genetic and biochemical analyses and blinding is not a factor of these studies s there is no operator bias.

Reporting for specific materials, systems and methods

We require information from authors about some types of materials, experimental systems and methods used in many studies. Here, indicate whether each material, system or method listed is relevant to your study. If you are not sure if a list item applies to your research, read the appropriate section before selecting a response.

Materials & experimental systems

n/a	Involved in the study	
☐	☒	Antibodies
☒	☐	Eukaryotic cell lines
☒	☐	Palaeontology and archaeology
☐	☒	Animals and other organisms
☒	☐	Human research participants
☒	☐	Clinical data
☒	☐	Dual use research of concern

Methods

n/a	Involved in the study	
☒	☐	ChIP-seq
☒	☐	Flow cytometry
☒	☐	MRI-based neuroimaging

Antibodies

Antibodies used	Polyclonal antiserum to His-BcpT was generated in this study by Lampire Biologicals in a rabbit to the His-tagged version of BcpT and affinity purified to the His-tagged protein. These are not commercially available.<br>Polyclonal antibodies to the O-antigen of P. vulgatus strain CL10 generated in rabbits was described and validated in a previous study and are not commercially available. Goat anti-rabbit IgG alkaline phosphatase conjugated antibody was purchased from Thermo Scientific (Pierce #31340) and Goat anti-rabbit HRP conjugate was purchased from BioRad product number 170-6515.
Validation	Specificity of these antibodies was confirmed by western blot analysis using mutants deleted for genes that synthesize these molecules.

Animals and other organisms

Policy information about studies involving animals; ARRIVE guidelines recommended for reporting animal research

Laboratory animals	Rabbit
Wild animals	A single rabbit was immunized with His-BcpT for antiserum generation. This was performed at Lampire Biologicals
Field-collected samples	*For laboratory work with field-collected samples, describe all relevant parameters such as housing, maintenance, temperature, photoperiod and end-of-experiment protocol OR state that the study did not involve samples collected from the field.*
Ethics oversight	*Identify the organization(s) that approved or provided guidance on the study protocol, OR state that no ethical approval or guidance was required and explain why not.*

Note that full information on the approval of the study protocol must also be provided in the manuscript.
