# Supplementary material for: A proteolytically activated antimicrobial toxin encoded on a mobile plasmid of Bacteroidales induces a protective response
Source: Nat Commun. 2022 Jul 23;13:4258. doi: 10.1038/s41467-022-31925-w (PMC9308784; doi:10.1038/s41467-022-31925-w)
Supplement: Supplementary file 3 — Description of Additional Supplementary Files [file 41467_2022_31925_MOESM3_ESM.pdf]

Title: Supplementary Data 1
Description: Tab 1 - edgeR and DESeq2 analyses of DGE of PvCL10 treated with BcpT for 3 hours versus untreated PvCL10 (columns L, M, O, P), or PvCL10. Genes highlighted meet the arbitrarily assigned cut-off for being classified as differentially expressed for each condition. Gene designations are based on NCBI
Supplementary Data 1 - Tab 2 - Top 100 most up and down DEGs when PvCL10 is treated with BcpT for 3 hours based on DESeq2 padj value. Gene of BvATCC that has the reciprocal best hit (RBH) to the BvCL10 gene and its DGE (up, down or no significant change) when treated with bacteroidetocin A (as reported in Matano et al. mBio. 2021 Oct 26;12(5):e0228521)

Title: Supplementary Data 2
Description: Tab 1 - Alignments of pBCPT plasmid backbone and bcpT in Bacteroidales genomes. Supplementary Data 2. Tab 2 - Presence of BcpT orthologs encoded by Bacteroidales genomes.

Title: Supplementary Data 3.
Description: Human gut metagenomic samples, of 1767 total samples, where bcpT was detected, correlated with abundances of P. vulgatus and P. dorei. Shading represents values passing indicated cutoff values.
